# Repeatability of AI-quantified incidental findings on lung cancer screening CT scans in the NELSON trial

**DOI:** 10.1007/s00330-026-12669-3

**Published:** 2026-06-17

**Authors:** Stijn Bunk, Thijs Bruins Slot, Edwin Bennink, Grigory Sidorenkov, Nils van der Velden, Niels Schurink, Félix Lades, Markus Sebald, Marjolein A. Heuvelmans, Hester A. Gietema, Joachim G. Aerts, Geertruida H. de Bock, Cornelia Schaefer-Prokop, Pim A. de Jong, Rozemarijn Vliegenthart, Firdaus A. A. Mohamed Hoesein, NELSON-POP consortium, Stijn Bunk, Grigory Sidorenkov, Marjolein A. Heuvelmans, Hester A. Gietema, Joachim G. Aerts, Cornelia Schaefer-Prokop, Pim A. de Jong, Rozemarijn Vliegenthart, Firdaus A. A. Mohamed Hoesein, Robin Cornelissen, Ralph Stadhouders, Jeroen G. J. van Rooij, Lianne Trap, Kristiaan Nackaerts, Walter de Wever, Mathias Prokop, Colin Jacobs, Noa Antonissen, Geertruida H. de Bock, Danrong Zhong, Harry J. M. Groen, Nils van der Velden, George S. Downward, Roel C. H. Vermeulen

**Affiliations:** 1 University Medical Center Utrecht, Utrecht University, Utrecht, The Netherlands; 2 University Medical Center Groningen, University of Groningen, Groningen, The Netherlands; 3 Siemens Healthineers Nederland B.V, Zoetermeer, The Netherlands; 4 Siemens Healthineers AG, Forchheim, Germany; 5 Maastricht University Medical Center, Maastricht University, Maastricht, The Netherlands; 6 University Medical Center Rotterdam, Erasmus University Rotterdam, Rotterdam, The Netherlands; 7 Radboud University Medical Center, Nijmegen, The Netherlands; 8 University Hospital Leuven, Catholic University of Leuven, Leuven, Belgium

**Keywords:** Artificial intelligence, Coronary artery disease, Emphysema, Thoracic vertebrae, Tomography (x-ray computed)

## Abstract

**Objectives:**

Computed tomography (CT) scans for lung cancer screening provide the opportunity of quantifying incidental findings. We evaluated the repeatability of AI-based measurements of incidental findings using short-term repeat CT scan pairs.

**Materials and methods:**

AI-Rad Companion Chest CT software was applied to low-dose non-contrast CT scans from the NELSON lung cancer screening trial to measure aorta diameters, coronary artery calcium volume (CACV), vertebral height and radiodensity, and emphysema (low attenuation area percentage, LAA%). Categories (absent/present) of aortic dilatation and osteopenia, and severity (none/mild/moderate/severe) of CACV and emphysema were calculated. We included subjects who had a short-term repeat CT scan pair with a maximum interval of 120 days. We analyzed repeatability with absolute and relative differences, and agreement with the intraclass correlation coefficient (ICC) and Cohen’s kappa.

**Results:**

1436 subjects were included, with age (mean ± SD) 59.7 ± 5.7 years, 86.3% men, 55.9% currently smoking, and scan interval 85 ± 20 days. Mean absolute differences were 0.7 to 1.5 mm for aorta diameters, 26 mm^3^ for CACV, 0.3 mm to 0.4 mm for vertebral height, and 7.6 HU for vertebral radiodensity. Median absolute difference was 0.8% for LAA%. Aorta diameters showed good (0.75 < ICC < 0.9) to excellent (ICC > 0.9) agreement. Agreement for the rest was excellent. Categorization Cohen’s kappa between the first and second measurements was 0.68 for aortic dilatation, 0.63 for CACV, 0.84 for osteopenia, and 0.70 for emphysema.

**Conclusion:**

In a lung cancer screening cohort with short-term repeat CT, the repeatability and agreement of automated AI measurements of aortic diameters, coronary calcium, vertebral height and radiodensity, and emphysema was good to excellent.

**Key Points:**

***Question*** *Can AI measurements of incidental findings beyond lung nodules be repeatably performed on pairs of lung cancer screening chest CT scans of the same subject?*

***Findings*** *The repeatability and agreement of AI-based measurements of aorta diameter, coronary calcium, vertebrae, and emphysema were generally excellent.*

***Clinical relevance*** *Measurement by radiologists of incidental findings on lung cancer screening chest CT scans imposes a high workload. AI algorithms allow for automatic measurement of incidental findings on lung cancer screening chest CT with high repeatability and agreement.*

**Graphical Abstract:**

**Figure:**
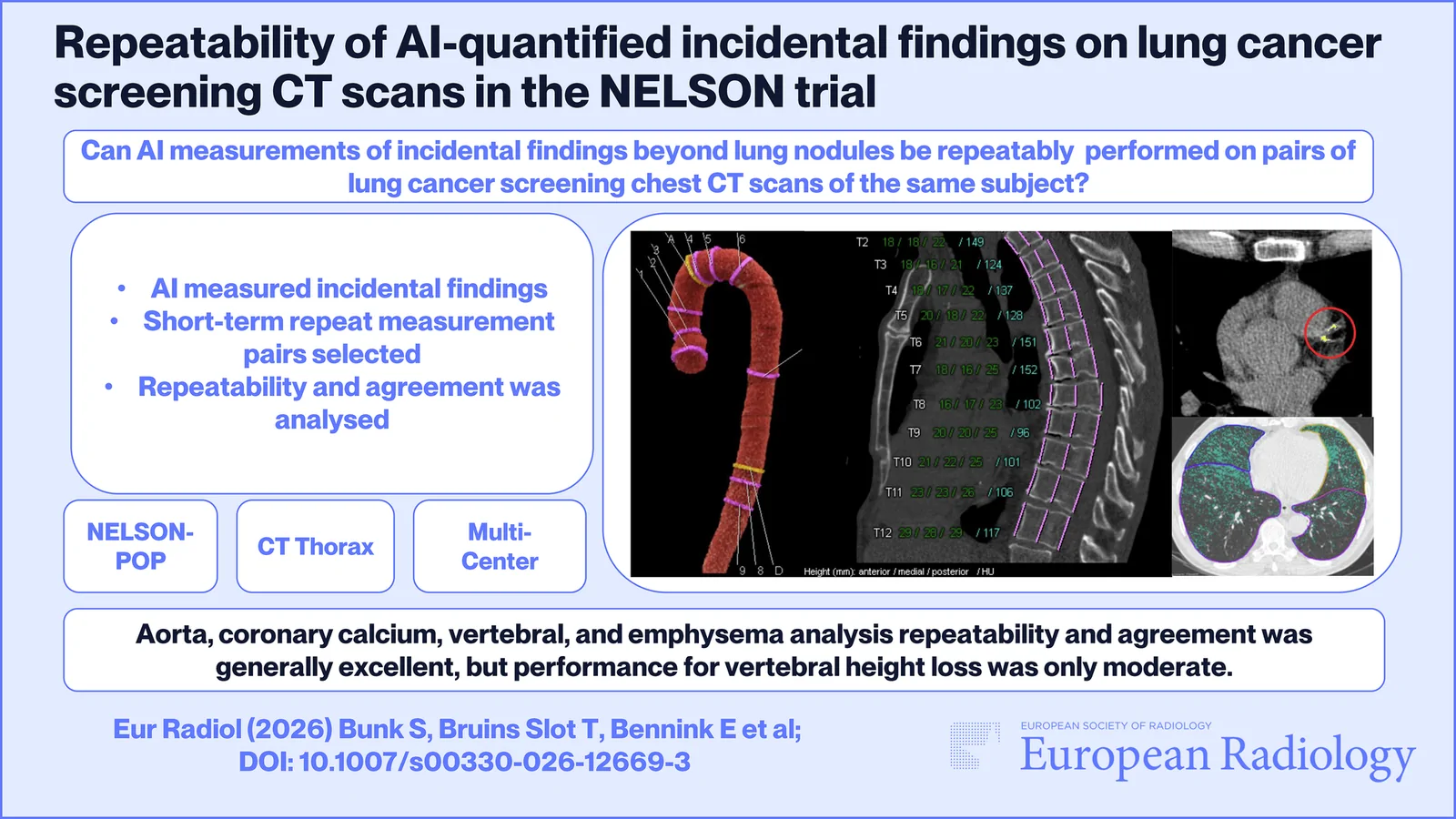

## Introduction

Since multiple trials demonstrated a significant reduction in lung cancer mortality from screening with low-dose chest computed tomography (CT), lung cancer screening has been implemented in several countries [1, 2]. This has created an opportunity to analyze incidental findings, such as cardiovascular calcifications, aortic dilatation, emphysema, early interstitial lung disease and osteopenia [3–7].

Although these incidental findings can predict clinically relevant events and hold promise for further gains in morbidity and mortality reduction in persons who smoke or have smoked, they further increase the workload of radiologists [8, 9]. This workload may hamper the feasibility of screening and, in addition, could lead to an increase in interpretive errors [10, 11]. Artificial intelligence (AI) methods may enable the automated measurement of incidental findings other than lung nodules on chest CT scans with a lesser increase in the workload of radiologists [12]. Previous studies found that AI algorithms can achieve good accuracy in measuring coronary calcium and aortic dilation on non-contrast-enhanced chest CT scans [13].

Before implementing AI for the measurement of incidental findings in lung cancer screening, several metrics need to be assessed. In addition to accuracy, the repeatability and agreement of measurements are a crucial performance metric [14]. It is important to be able to distinguish whether an abnormal value or change of a measurement may be the result of measurement variability or is an actual abnormality or change.

Prior studies on the validation of AI measurements on ungated non-contrast low-dose chest CT exist. For aorta diameter measurements, Hamelink et al showed mean absolute differences between a manual reader and AI-Rad Companion Chest CT lower than 2 mm for all measurement positions in 240 subjects [15]. For CACV, van Assen et al demonstrated excellent correlation between manual scoring on cardiac CT and AI-based coronary artery calcium measurements on chest CT in 263 subjects [16]. In a different study by Hamelink et al, the repeatability of AI-Rad Companion Chest CT incidental finding measurements was investigated in 632 participants of the ImaLife study with a 3–4 month scan interval [14]. They found that measurements of vertebral height and radiodensity and aortic diameters showed excellent repeatability and that CACV risk categorization showed high concordance. However, the scanning concerned a specific, high-pitch technique; results have not been validated in a lung cancer screening setting.

Therefore, the aim of this study was to assess the repeatability and agreement of measurements of various potentially relevant incidental findings measured by AI software on short-term follow-up low-dose lung cancer screening scan pairs from the Dutch-Belgian Randomized Lung Cancer Screening (NELSON) Trial.

## Methods

### Study population

On December 23, 2003, the Minister of Health of the Netherlands approved randomization of persons to the NELSON trial. On March 22, 2006, the trial was retrospectively registered in the Overview of Medical Research in the Netherlands (OMON) (NL-OMON22971). Written informed consent was obtained from all participants.

The current analysis is part of the NELSON-POP project, which aims to optimize participant selection and nodule management in lung cancer screening using a multimodal data approach [17]. The data used in this retrospective study were obtained in the NELSON trial. The NELSON trial included individuals aged 50–75 years with significant smoking history, scanned at the University Medical Center Groningen, the University Medical Center Utrecht, the Kennemer Gasthuis Haarlem, all in the Netherlands, and the University Hospitals Leuven, Belgium. Detailed inclusion and exclusion criteria of the NELSON trial have previously been described [18–20]. The current study comprised Dutch participants only. In the NELSON trial, some subjects received short-term follow-up CT scans. The reason for a short-term follow-up CT scan was for follow-up of an indeterminate lung nodule.

### Image acquisition protocol

Non-contrast low-dose chest CT scans were obtained in inspiration with 16-slice multidetector CT systems with collimation of 16 × 0.75 mm (Sensation 16, Siemens AG Medical Solutions; M × 8000 IDT, and Brilliance 16 P, Philips Medical Systems). The settings used were fixed 30 mAs with 120 kVp for participants weighing 80 kg or less and 140 kVp for those weighing more than 80 kg. Medium-soft reconstruction kernels (Philips B, Siemens B30f) were used to reduce noise. Data were reconstructed as axial slices of 1.0 mm at 0.7 mm increment [6, 19, 21, 22]. Other than repeat scans sometimes using a different kVp, the same scanner and acquisition and reconstruction parameters were used for each scan of each subject. Details on the scan kVp distribution are provided in Appendix A.

### Automated scan analysis

Measurements were performed on all scans from the Dutch NELSON trial sites satisfying the requirements of the AI software, Siemens Healthineers’ AI-Rad Companion Chest CT (On-premises prototype version 0.5.0.6 for emphysema measurement, Cloud product version VA40 for all other measurements, Siemens Healthineers). For the emphysema quantification, a special filtering was implemented in the Siemens Healthineers’ AI-Rad Companion Chest CT on-premises prototype version 0.5.0.6 to improve performance on low-dose data. To deal with high noise levels, a connected component clustering using a 3D-26 voxel neighborhood was employed, filtering only components including at least 50 voxels.

The aortic diameters were measured at multiple predefined locations. The extent of coronary calcification was assessed as total Coronary Artery Calcium Volume (CACV). Anterior, medial, and posterior height, as well as mean radiodensity, were measured for each vertebral body. Emphysema was quantified as low attenuation area percentage (LAA%, percentage of lung voxels below −950 HU). Before a final dataset of all measurements was created, a subset of the AI-generated segmentations, from which the measurements were derived, was checked for errors, as described in Appendix B. In short, for each type of measurement, a selection of possibly erroneous measurements was made based on a method appropriate for each type of measurement. These measurements were visually checked by a reader under supervision by a board-certified EBCR radiologist with 15+ years of experience. Measurements derived from a visually assessed inadequate segmentation were excluded from the dataset. This yielded a dataset with measurements that were successfully processed by the AI software.

### Data selection for repeatability and agreement analysis

Because only short-term follow-up scan measurement data is suitable for the repeatability and agreement analysis, we selected only the measurements made on pairs of scans that were made at most 120 days apart. We define the baseline measurements to be the measurements made on the baseline scan (first scan of the scan pair) and the repeat measurement to be the measurements made on the repeat scan (second scan of the scan pair). The baseline scan was not necessarily the first scan of a screening participant, but any screening round scan after which a short-term follow-up scan was performed. If multiple short-term follow-up scan pairs were available for a participant, only the first pair of scans was included. Therefore, each participant was included only once in the analysis.

### Categorization of measurements

Based on accepted criteria as well as measurements used in the National Lung Screening in the United Kingdom, we calculated various derived categorical measures [5, 23–25].

CACV and LAA% were divided into four categories: none, mild, moderate, and severe. For CACV, it was defined as none: 0 mm^3^, mild: > 0–100 mm^3^, moderate: > 100–300 mm^3^, and severe: ≥ 300 mm^3^ [5]. For LAA%, it was defined as none: < 5%, mild: ≥ 5 to < 10%, moderate: ≥ 10 to < 15%, and severe: ≥ 15% [5].

The maximum ascending aorta diameter and mean vertebral radiodensity per subject were divided into two categories. Based on the maximum ascending aorta diameter, we defined ≤ 45 mm as no dilatation, and > 45 mm as dilatation based on the UK criteria [5]. Osteopenia categories were based on mean vertebral radiodensity: ≤ 115 HU indicated osteopenia, and > 115 HU indicated no osteopenia based on previous publications [5, 25–28].

### Statistical analysis

Continuous variables related to vertebral, aortic, and emphysema measurements are reported as mean ± standard deviation (SD). Due to non-normal distribution, CACV and LAA% are reported as median and interquartile range (IQR).

Repeatability of baseline and repeat measurements was evaluated using the absolute and relative difference between baseline and repeat measurements, as well as the within-subject standard deviation and repeatability coefficient [29, 30]. Relative difference was defined as ((100 × |baseline – repeat|) / mean of baseline and repeat).

Agreement between baseline and repeat measurements was evaluated using the intraclass correlation coefficient (ICC) with corresponding 95% confidence intervals (CI). We used ICC(A,1). ICC values were interpreted as follows: > 0.90, excellent; 0.75–0.90, good; 0.50–0.75, moderate; and < 0.50, poor agreement [31].

The calculated categorical variables were compared between baseline and repeat measurements. We determined concordance percentages with a confusion matrix. We also determined agreement of absent/present categorization via an unweighted Cohen’s kappa and agreement of severity categorization via a quadratically weighted Cohen’s kappa.

Statistical analyses were performed using Python, R 4.4.1, and the R packages *irr* and *yardstick* [32–34].

## Results

**Fig. 1:**
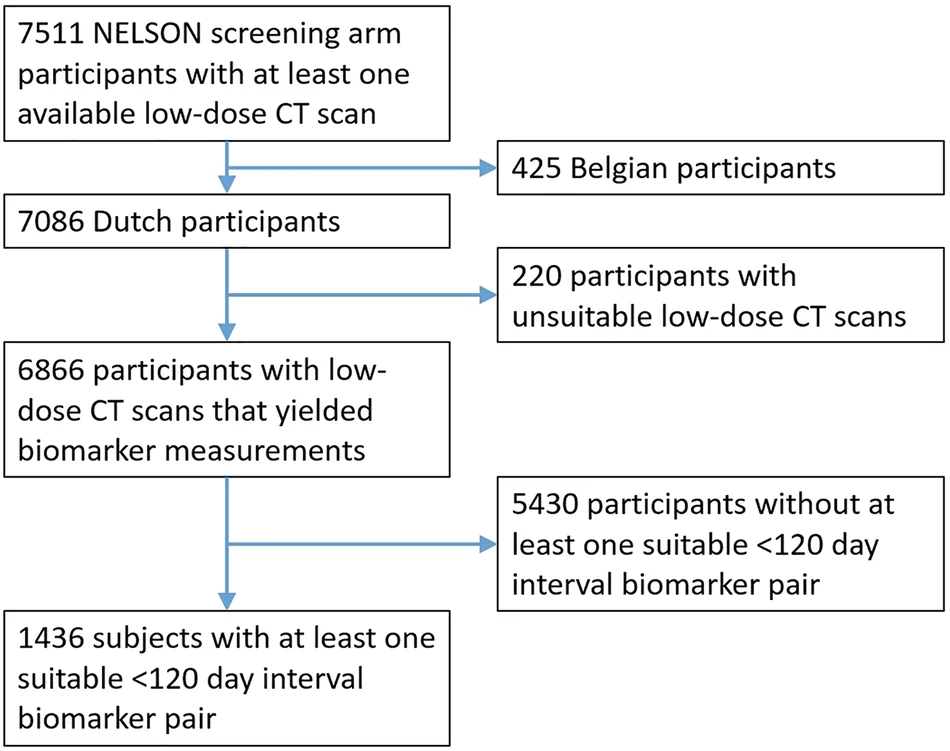
Subject selection of flowchart. Only analyses based on the 1436 subjects are presented in this paper

**Fig. 2:**
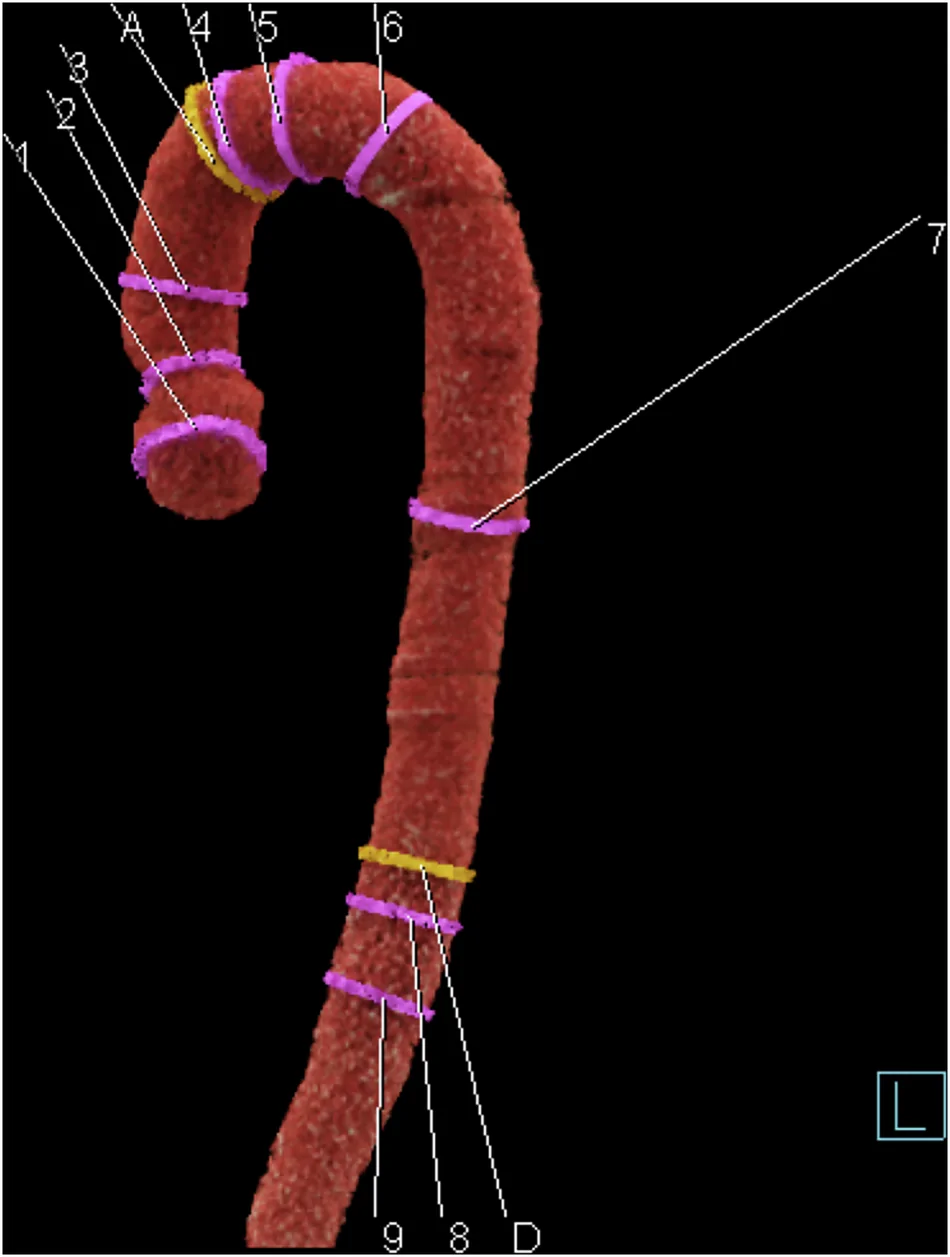
Example of aorta segmentation and diameter measurement locations. 1: Sinus of Valsalva, 2: Sinotubular junction, 3: Mid ascending aorta, 4: Proximal aortic arch, 5: Mid aortic arch, 6: Proximal descending aorta, 7: Mid ascending aorta, 8: Aorta at diaphragm, 9: Aorta at celiac artery origin, A: Maximum ascending aorta diameter, D: Maximum descending aorta diameter

**Fig. 3:**
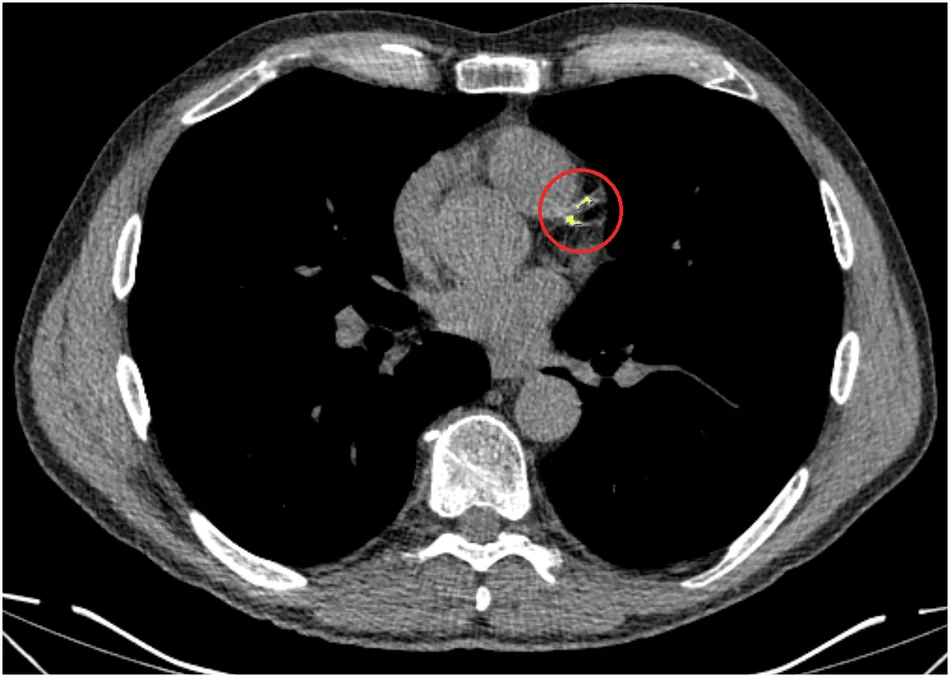
Example segmentation of coronary calcium. The red circle indicates the coronary calcium location. Coronary calcium is displayed in yellow

**Fig. 4:**
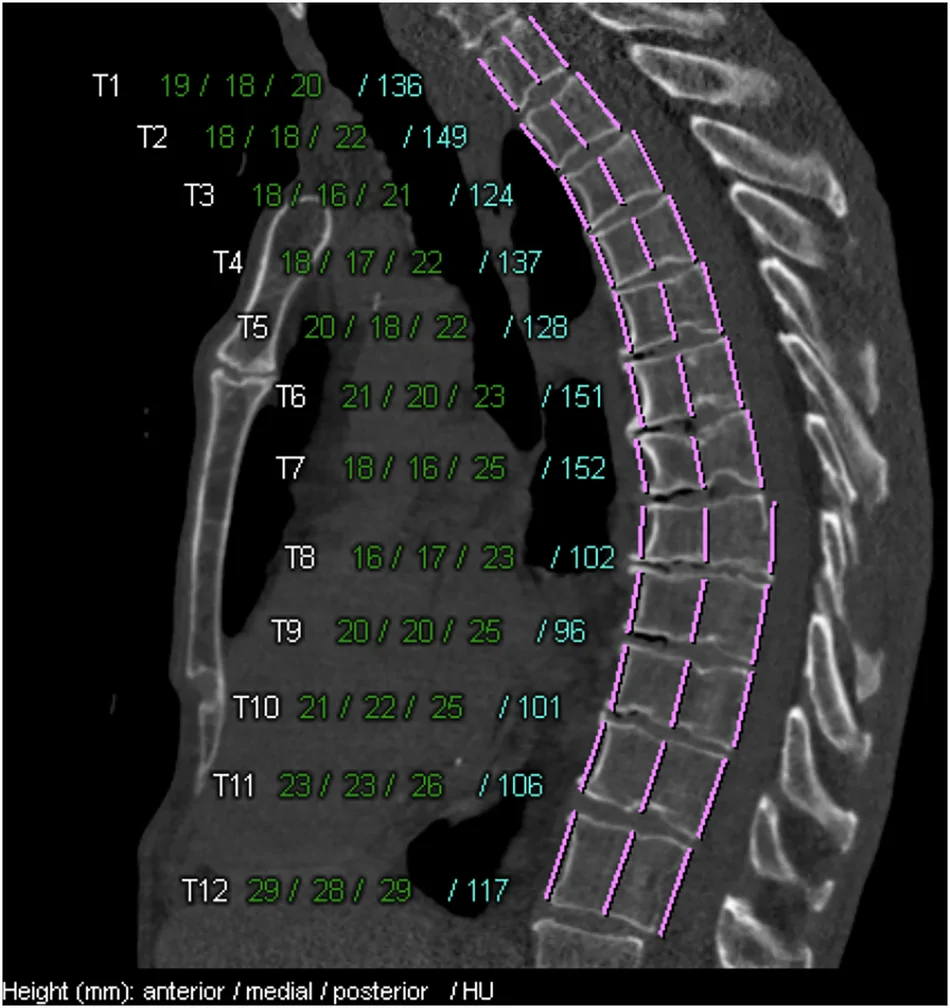
Example of vertebral height and density measurements. From left to right, the labels indicate the vertebrae (T2 = 2nd thoracic vertebrae), the anterior height, the medial height, the posterior height (all in mm), and the radiodensity (in HU). The pink lines are the measurement locations, from left to right: anterior, medial, and posterior

**Fig. 5:**
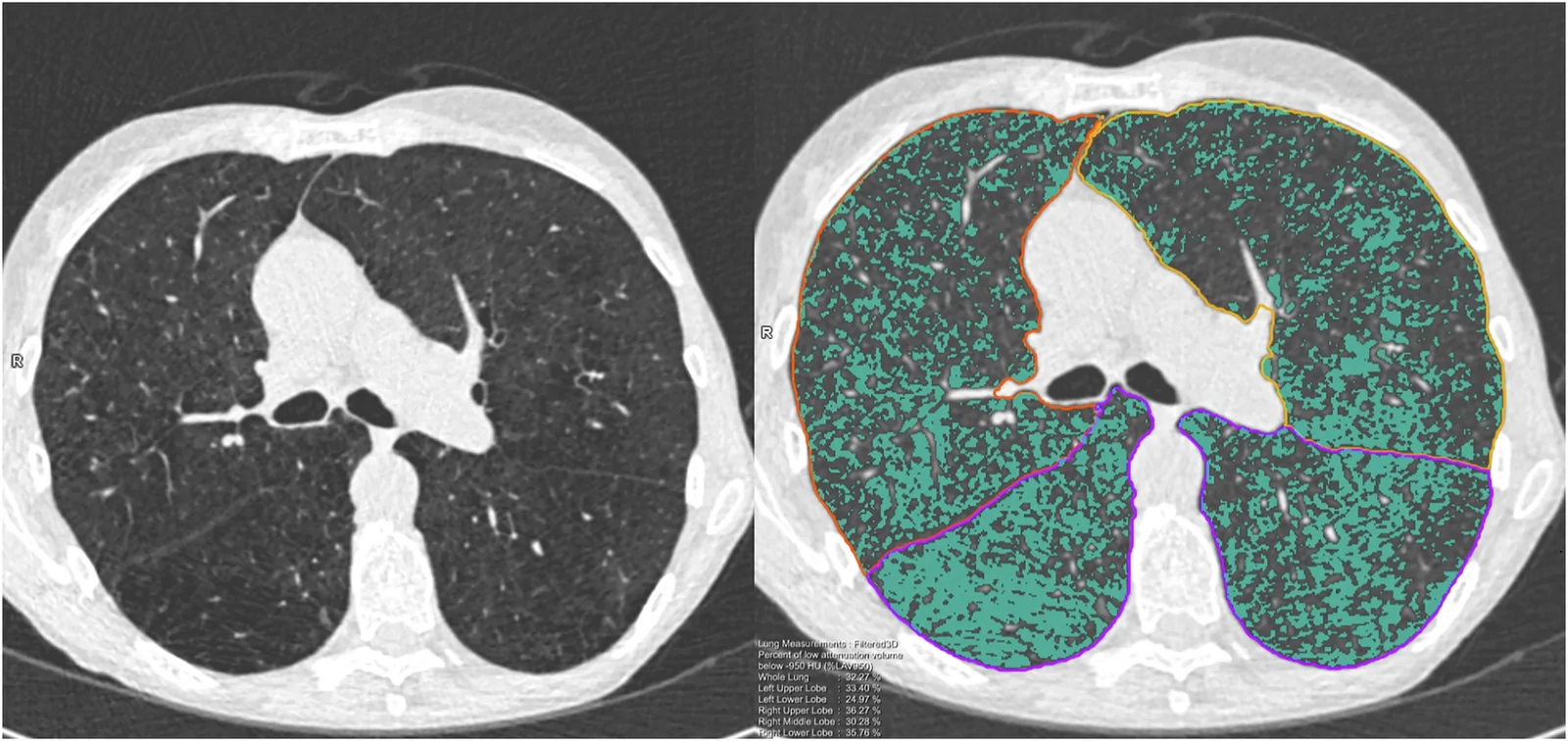
Example of subject with a high amount of emphysema. Left: CT slice. Right: emphysema segmentation, displayed in cyan. Colored outlines indicate lung lobes

1436 subjects (mean ± SD age 59.7 ± 5.7 years, 86.3% men, 55.9% currently smoking) had at least one pair of baseline and short-term repeat measurements from CT scans made within 120 days of each other (mean ± SD interval 85 ± 20 days). The subject selection process is shown in Fig. 1. Examples of the segmentations of each measurement type are given in Figs. 2–5.

Full details on the baseline and repeat measurements and categorization-based subclinical disease presence are given in Table 1. Based on the error check described in Appendix B some measurements were excluded. The number of excluded measurement pairs is also shown in Table 1. Reasons for measurement exclusion were varied and included problems beyond algorithm failure, such as inadequate image quality or prior surgical intervention. In Appendix C some examples of correct and incorrect measurements are shown.

**Table 1:** Baseline and repeat measurement characteristics

Measurement type	Baseline	Repeat	Excluded pairs
Aorta diameters			
Maximum of ascending aorta mm (*n* = 1427)	38.4 ± 3.9	38.4 ± 3.8	9 (0.6%)
Maximum of descending aorta mm (*n* = 1427)	31.6 ± 3.2	31.6 ± 3.2	9 (0.6%)
Sinotubular junction mm (*n* = 1428)	33.7 ± 3.7	33.7 ± 3.8	8 (0.6%)
Mid ascending aorta mm (*n* = 1428)	36.8 ± 4.0	36.7 ± 3.9	8 (0.6%)
Proximal aortic arch mm (*n* = 1429)	34.2 ± 3.2	34.1 ± 3.2	7 (0.5%)
Mid aortic arch mm (*n* = 1429)	31.2 ± 2.7	31.2 ± 2.7	7 (0.5%)
Proximal descending thoracic aorta mm (*n* = 1429)	29.6 ± 2.9	29.6 ± 3.0	7 (0.5%)
Mid descending thoracic aorta mm (*n* = 1428)	28.0 ± 2.7	27.9 ± 2.7	8 (0.6%)
Aorta at diaphragm mm (*n* = 1376)	27.4 ± 2.6	27.4 ± 2.6	60 (4.2%)
Aortic dilatation % (> 45 mm) (*n* = 1427)	4.2	3.6	9 (0.6%)
Coronary calcium			
CACV mm^3^ (*n* = 1367)	76.2 (5.8–310.1)	75.8 (8.1–314.0)	69 (4.8%)
Moderate or severe CACV (> 100 mm^3^) (*n* = 1367) (%)	45.2	45.4	69 (4.8%)
Vertebrae			
Anterior height mm (*n* = 1421)	20.1 ± 1.2	20.1 ± 1.2	15 (1.0%)
Medial height mm (*n* = 1421)	19.4 ± 1.2	19.4 ± 1.2	15 (1.0%)
Posterior height mm (*n* = 1421)	22.1 ± 1.3	22.1 ± 1.3	15 (1.0%)
Radiodensity HU (*n* = 1421)	161.9 ± 38.5	161.1 ± 38.0	15 (1.0%)
Osteopenia % (< 115 HU) (*n* = 1421)	10.3	10.5	15 (1.0%)
Emphysema			
Emphysema LAA% (*n* = 1341)	3.8 (1.2–9.0)	3.9 (1.2–9.6)	95 (6.6%)
Moderate or severe emphysema % (> 10%) (*n* = 1323)	20.5	22.5	95 (6.6%)

Values in the “Baseline” and “Repeat” columns are reported as mean ± standard deviation, median (interquartile range), or percentage. Values in the “Excluded pairs” column indicate how many measurements were excluded due to the error check, described in Appendix B, with the percentage relative to the 1436 subjects in brackets

*CACV* coronary artery calcium volume, *HU* Hounsfield unit, *LAA%* low attenuation area percentage

Table 2 shows the absolute and relative differences between the baseline and repeat measurements for each incidental finding. For most incidental findings, the mean absolute and relative differences were < 5%. There were a few exceptions, specifically CACV and LAA%, which had median relative differences above 25%.

**Table 2:** Repeatability and agreement of measurements

Measurement type	Absolute difference	Relative difference (%)	Within-subjects SD	RC	ICC
Aorta diameters					
Maximum of ascending aorta mm (*n* = 1427)	0.8 ± 1.0	2.2 ± 2.5	0.9 (0.9–1.0)	2.5	0.943 (0.937–0.949)
Maximum of descending aorta mm (*n* = 1427)	0.7 ± 0.8	2.1 ± 2.6	0.8 (0.7–0.8)	2.1	0.946 (0.940–0.951)
Sinotubular junction mm (*n* = 1428)	1.5 ± 1.5	4.6 ± 4.4	1.5 (1.5–1.6)	4.2	0.837 (0.821–0.852)
Mid ascending aorta mm (*n* = 1428)	0.9 ± 1.0	2.5 ± 2.7	0.9 (0.9–1.0)	2.6	0.944 (0.938–0.949)
Proximal aortic arch mm (*n* = 1429)	1.0 ± 1.0	2.8 ± 3.0	1.0 (1.0–1.0)	2.8	0.906 (0.896–0.915)
Mid aortic arch mm (*n* = 1429)	0.8 ± 0.8	2.5 ± 2.7	0.8 (0.8–0.8)	2.2	0.912 (0.903–0.920)
Proximal descending thoracic aorta mm (*n* = 1429)	0.8 ± 1.0	2.8 ± 3.4	0.9 (0.9–0.9)	2.5	0.908 (0.898–0.916)
Mid descending thoracic aorta mm (*n* = 1428)	0.7 ± 0.8	2.3 ± 2.9	0.7 (0.7–0.8)	2.0	0.926 (0.918–0.933)
Aorta at diaphragm mm (*n* = 1376)	0.9 ± 0.9	3.2 ± 3.3	0.9 (0.9–0.9)	2.5	0.878 (0.866–0.890)
Coronary calcium					
CACV mm^3^ (*n* = 1367)	26.0 (5.8–90.9)	29.5 (7.2–79.6)	121.7 (117.1–126.2)	337	0.931 (0.923–0.938)
Vertebrae					
Anterior height mm (*n* = 1421)	0.4 ± 0.3	1.8 ± 1.6	0.3 (0.3–0.4)	0.9	0.914 (0.905–0.922)
Medial height mm (*n* = 1421)	0.3 ± 0.3	1.5 ± 1.4	0.3 (0.3–0.3)	0.8	0.948 (0.942–0.953)
Posterior height mm (*n* = 1421)	0.4 ± 0.4	1.7 ± 1.6	0.4 (0.3–0.4)	1.0	0.927 (0.919–0.934)
Radiodensity HU (*n* = 1421)	7.6 ± 9.4	4.8 ± 5.6	8.5 (8.2–8.8)	23.6	0.950 (0.945–0.955)
Emphysema					
Emphysema LAA% (*n* = 1341)	0.8 (0.2–2.1)	25.3 (10.3–50.1)	1.6 (1.6–1.7)	4.5	0.942 (0.935–0.948)

Absolute and relative differences are reported as mean ± standard deviation or median (interquartile range). Within-subjects standard deviation (SD) is reported as SD (95% confidence interval). Intraclass correlation coefficient (ICC) is reported as ICC (95% confidence interval)

*RC* repeatability coefficient, *CACV* coronary artery calcium volume, *HU* Hounsfield unit, *LAA%* low attenuation area percentage

Additionally, in Table 2, the ICC for all continuous measurements is shown. Out of the sixteen groups of measurements, agreement was excellent (ICC > 0.9) for 13 measurements and good (0.75 < ICC < 0.9) for two measurements, both regarding aorta diameter.

In Table 3, Cohen’s kappa is shown for each categorized incidental finding. Categorization of aortic dilatation, CACV, osteopenia, and LAA% all showed substantial agreement between the baseline and repeat measurements. Overall concordance for all measures was above 70%. For the incorrect classifications, 99% were assigned to the nearest adjacent category.

**Table 3:** Cohen’s Kappa for categorized incidental findings

Measurement	Presence kappa	Severity kappa
Aortic dilatation presence	0.68	N/A
CAC presence and severity	0.83	0.87
Osteopenia presence	0.84	N/A
Emphysema presence and severity	0.77	0.89

The presence kappa is unweighted and based on absent/present categories, which are the only categories available for aortic dilatation and osteopenia. For CAC presence and severity and emphysema presence and severity, the Presence kappa is based on two groups: none/mild and moderate/severe. The Severity kappa is quadratically weighted and based on the four individual severity categories of none, mild, moderate, and severe

*CAC* coronary artery calcium

Full details on the vertebral measurements at each level are given in Appendix D. In Appendix E an analysis of vertebral height loss is performed. In Appendix F the confusion matrices for maximum ascending aorta diameter, CACV, vertebral radiodensity, vertebral height loss, and emphysema LAA% are shown.

### Aorta

The mean absolute difference of the aorta diameter measurements was very low, with only the absolute sinotubular junction diameter difference exceeding 1 mm. For the location-based aorta measurements, agreement was lowest at the sinotubular junction with an ICC of 0.832 (95% CI: 0.815–0.847) and highest for the mid descending thoracic aorta diameter with an ICC of 0.926 (95% CI: 0.918–0.933). The maximum ascending aorta diameter had the second highest agreement with an ICC of 0.943 (95% CI: 0.937–0.949).

Aortic dilatation (diameter > 45 mm) was present in 4.2% of 1427 subjects at baseline. Aortic dilatation presence had a Cohen’s kappa of 0.68, with an overall concordance of 97.6%.

### CACV

For CACV, the median absolute difference was 26.0 (IQR: 5.8–90.9) and the median relative difference was 29.5% (IQR: 7.2–79.6), while ICC agreement was excellent with an ICC of 0.931 (95% CI: 0.923–0.938).

Moderate or severe coronary calcium (CACV > 100 mm^3^) was present in 45.2% of 1367 subjects at baseline. CACV severity categorization had a Cohen’s kappa of 0.87, with an overall concordance of 72.6%.

### Vertebrae

For the vertebral measurements, the mean absolute differences for the height measurements were all ≤ 1 mm, with a mean radiodensity difference of 7.6 HU. For the vertebral heights, agreement was excellent: an ICC of 0.913 for the anterior height (95% CI: 0.904–0.921) and an ICC of 0.943 for the medial height (95% CI: 0.937–0.949).

On the baseline measurements, osteopenia (radiodensity ≤ 115 HU) was present in 10.4% of 1421 subjects. Osteopenia’s presence had a Cohen’s kappa of 0.84, with an overall concordance of 96.8%.

### Emphysema

The median absolute difference in LAA% was 0.8%, but the relative difference was 25%. The IQR of the absolute differences was 0.2–2.1%, but the IQR of the relative difference was 10.3–50.1%. Agreement was excellent for LAA% with an ICC of 0.942 (95% CI: 0.935–0.948).

Moderate or severe emphysema (LAA% > 10%) was present in 20.5% of 1323 subjects at baseline. LAA% severity categorization had a Cohen’s kappa of 0.89, with an overall concordance of 82.3%.

## Discussion

AI-based measurements of potentially relevant incidental findings in low-dose CT for lung cancer screening may hold promise to improve the outcome and benefits of screening without increasing the workload for radiologists. However, knowledge of the performance of automatic analyses is crucial, both to determine the probability of a finding being abnormal and to allow for accurate assessment of progression. Therefore, we assessed the repeatability and agreement of various potentially relevant incidental findings measured by AI-Rad Companion Chest CT on baseline and short-term repeat non-contrast low-dose chest CT scans from lung cancer screening participants in the NELSON trial. We found that measurement of aortic dilatation, coronary calcium severity, emphysema severity, osteopenia, and vertebral height had high repeatability and agreement.

Even though the LDCT scans of the NELSON cohort were performed with older CT technology, without the use of iterative reconstruction, overall, the repeatability of state-of-the-art AI quantification was high. For CACV and LAA%, the agreement was excellent, but the median relative differences were high. For CACV and LAA%, the high median relative differences are likely caused by the low end of the measurements being close to zero, and thus any small change in measurement value will result in a large relative change. For example, going from 3 mm^3^ of CACV to 9 mm^3^ of CACV is a 200% relative difference. However, the repeatability coefficient was also high for these measurements, indicating that there is a higher degree of measurement variability. A possible cause for higher variability for CACV could be the ungated nature of the scans, as that may introduce movement artifacts. For LAA%, it could be the low-dose nature of the scans, which increases image noise.

For risk categorization based on decision thresholds, high concordance and agreement were achieved for all incidental findings. Additional analyses on the impact of incorrect classifications showed that in 99% of cases, incorrect classifications were assigned to adjacent categories, limiting the effect of wrong classifications on referral decisions. Additionally, the repeatability coefficients indicate that clinically significant differences for aortic dilatation, vertebral height, and vertebral radiodensity are small relative to the mean of the population. However, for CACV and LAA%, the high repeatability coefficients indicate that clinically significant differences may require a large change relative to the median of the population. However, this high repeatability coefficient may partially be caused by those measurements not being normally distributed, as the repeatability coefficient assumes the data is normally distributed.

Compared to the prior study by Hamelink et al, we found similar but slightly higher mean absolute and relative differences for the aorta diameters, CACV, and vertebral measurements [14]. However, they did not investigate emphysema. The lower repeatability in our study compared to the previous study by Hamelink et al is likely caused by the scans in the NELSON trial being acquired on significantly older CT scanners than those used in the ImaLife study. Acquisition of NELSON scans began in 2006, whilst ImaLife began in 2016. In the intervening decade, there were significant improvements to the quality of scans obtained on CT scanners. For example, in ImaLife, third-generation dual-source CT scanners (SOMATOM Force, Siemens) were used, which allow faster acquisition, and reconstruction was done via filtered back projection with a Br40 kernel [35]. If the scans to be processed are of lower quality, greater variability in the measurements can be expected. It is important to note that a low-dose acquisition protocol or lower quality scans do not affect all types of measurements equally. Measurements based on anatomical locations, such as the aorta diameters or vertebral heights, will see less increase in variability from reduced signal to noise in a scan compared to those measurements that more directly rely on the radiodensity of a voxel, such as emphysema.

The results of this study are of importance since lung cancer screening is being performed in many countries around the world. There is a debate on which incidental findings should be reported and acted on [3–5]. One of the primary issues, in addition to potential overdiagnosis and overtreatment, is the reading burden for the radiologist. Previous research has established that measurements made by AI algorithms closely match assessments by radiologists [15, 16]. We show that automated analyses of low-dose lung cancer screening scans by an AI algorithm, like AI-Rad Companion Chest CT, provide measurements with sufficient repeatability to determine clinically relevant differences and risk categorizations for aortic dilatation, coronary calcification severity, emphysema severity, and osteopenia. Moreover, we demonstrated this on ungated non-contrast low-dose CT scans acquired on multiple older CT scanner models using different reconstruction kernels and kVp settings, demonstrating AI algorithm robustness when processing non-ideal input data. On more modern data, better performance is expected, as was observed in the ImaLife study [14]. Therefore, reporting on incidental findings with AI assistance will incur little additional reading burden for radiologists. However, it is important to note that checking the validity of automated measurements and correcting errors is still required. The repeatability and agreement analyses described in this study were carried out after an error check was performed. This resulted in the exclusion of 0.5% to 6.6% of the short-term follow-up measurement pairs due to the exclusion of one or both data points, depending on the specific measurement. These error rates should not pose a significant problem if AI is to be implemented as a first or second reader in lung cancer screening, as having to correct only a minor number of measurements would still take less time per scan than manually measuring all incidental findings.

The major strength of our study was that our data included a large number of participants, who were acquired using a consistent scanning protocol across multiple centers and scanners as part of the NELSON trial. The NELSON trial is the second largest lung cancer screening trial so far, on which multiple currently ongoing lung cancer screening programs are based. This ensured our data is representative of lung cancer screening in general.

A limitation of our study was the lack of very short-term repeat scans, such as those acquired within 1 day. However, we do not expect there to be, on average, significant changes in the measured incidental findings in our 120-day window. A further limitation of our study was the usage of older CT data, potentially limiting the applicability of our conclusions to scans acquired on more modern scanners. However, it is important to note that current low-dose CT lung cancer screening protocols, such as that of NHS England, also use 16-slice MDCT scanner types and use similar radiation doses as the NELSON trial [5]. Furthermore, considering that CT scanners have improved in quality since the NELSON trial, repeatability is expected to improve on more modern scanners. The lack of benchmarks for emphysema measurement repeatability was also a limitation of our study. The assessment of repeatability of emphysema measurement on more modern scanners requires further investigation. Another limitation was that an error check had to be performed to exclude incorrect measurements. This means that in order to obtain similarly repeatable results, measurements must be checked for errors. Finally, the low proportion of women in our study was also a limitation. The original NELSON trial population included fewer women than men, and as our data derives from the NELSON trial, our population also includes fewer women than men.

In conclusion, the repeatability and agreement of repeat automated AI-based measurements of aortic dilatation, CACV severity, osteopenia, and emphysema severity in NELSON were good to excellent on these non-contrast low-dose CT screening scans acquired on older scanners. Further research should investigate the role of automatic measurements in currently ongoing lung cancer screening programs.

## Supplementary information

Supplementary information

## Abbreviations

CACV: Coronary artery calcium volume
ICC: Intraclass correlation coefficient
IQR: Interquartile range
LAA: Low attenuation area
SD: Standard deviation
