# Supplementary material for: Repeatability of AI-quantified incidental findings on lung cancer screening CT scans in the NELSON trial
Source: Eur Radiol. 2026 Jun 17;36(10):7524–34. doi: 10.1007/s00330-026-12669-3 (PMC13574856; doi:10.1007/s00330-026-12669-3)
Supplement: Supplementary file 1 [file 330_2026_12669_MOESM1_ESM.pdf]

Repeatability of AI-quantified incidental findings on lung cancer screening CT scans in the NELSON trial

ELECTRONIC SUPPLEMENTARY MATERIAL

Appendix A: Scan kVp distribution

Scan kVp	Number of scans at baseline	Number of scans at repeat
120 kVp	643	642
140 kVp	793	794

The scan kVp at baseline and repeat was the same in 1253 cases and different in 183 cases.

Appendix B: Error check and error exclusion protocol

The full dataset contained 6866 subjects and 25821 scans. To get an estimate of segmentation accuracy, and remove erroneous measurements, the segmentations of outlier measurements were reviewed for errors by a trained reader under supervision by a board-certified EBCR radiologist with 15+ years of experience. For each type of measurement (aorta, coronary arteries, vertebrae, and emphysema) the quality of measurement parameters was evaluated in a manner adjusted to that type of measurement. This resulted in a different error check strategy for each category of measurements. A common strategy was calculating the difference for each measurement from the leave-one-out mean of that measurement across all of a subject's scans. The left-out value was the measurement relative to which the difference to the mean was being calculated. Subjects or scans with the largest deviation(s) were then checked.

Besides erroneous measurements, we excluded the aortic diameter at the sinus of Valsalva and at the celiac artery origin from the analysis because of the ungated nature of the CT scans and high noise levels in the inferior section of the CT scans, respectively. Furthermore, we excluded the T1 and T12 vertebra levels from the analysis in view of the high noise levels at the superior and inferior regions of the scan volume.

Aorta

The quality of the aorta diameter measurements was assessed with a random sample and a selection of the largest diameter deviations. The random sample comprised 250 scan pairs, selected evenly from the Dutch participating centers. The scan pairs consisted of baseline scans paired with a short-term repeat scan (maximum interval 120 days), totaling 500 scans. Furthermore, the top 1% of deviating results (259 scans) were selected for a check. Deviation was determined by calculating the difference between a diameter measurement at a specific position and the leave-one-out mean of that diameter measurement across all of a subject's scans. In total, the aorta measurements were manually evaluated on 759 scans.

The error assessment was done by visually inspecting the 3D reconstruction of the aorta segmentation with an overlay of the aorta diameter measurements. Individual measurements were excluded from the dataset if segmentation was inaccurate or if they did not match the location/angulation prescribed in measurement guidelines.[1] If the majority of diameter measurements were considered incorrect, or if the aorta segmentation was insufficiently precise, causing multiple incorrect diameter measurements, then the full result was considered unusable and all measurements for a specific scan were excluded.

The aorta error check resulted in the exclusion of 120 diameter measurements from the dataset. Five scans had all 9 measurements excluded. For the individual exclusions, 17 measurements of the mid ascending aorta diameter, 24 measurements of the proximal aortic arch diameter, 23 measurements of the mid aortic arch diameter, 7 measurements of the proximal descending thoracic aorta diameter, and 4 measurements of the mid descending thoracic aorta diameter were excluded.

Coronary artery calcium volume

For coronary artery calcium volume (CACV) outliers were identified by two methods. The first method looked at the largest deviations (largest difference in CACV volume from the leave-one-out mean) and the second method looked at the highest CACV scores at baseline. The assumption for the first method was that large changes between scans were most likely to have been the result of an algorithm failure (like including mitral valve/pericardial calcifications), inconsistent imaging factors, or the presence of a coronary stent.

The CACV results were excluded from the dataset in six scenarios: the algorithm failed to include large coronary calcifications (>10% of the total volume), the algorithm incorrectly included large non-coronary regions as coronary calcification (>10% of the total volume), the CT images included excessive movement artifacts that greatly influenced the CACV, the subject had a coronary bypass or coronary stents, or other less common causes such as the presence of a pacemaker causing artefacts.

Axial maximum intensity projections of the scan were checked against an overlay of the coronary artery calcium (CAC) segmentation. In this view any large discrepancies between CAC and the AI segmentation were clearly visible, as well as excessive movement and artifacts. When in doubt (e.g. when the maximum intensity projection was unclear) the original thin-slice CT scan and segmentation overlay were inspected slice-by-slice to arrive at a conclusion.

Based on the first selection method of the largest deviations, 500 subjects with the largest CACV deviations across all repeat scans were selected for manual inspection, comprising 1863 scans. Also, the highest 1% of absolute CACV scores in baseline scans were selected for manual inspection. This corresponded to 38 scans which were not already included by the first outlier identification method. Thus, in total 1901 scans were visually checked.

In total 948 scans were determined to have unusable CACV measurements. The largest contribution to exclusion of results was the presence of coronary stents (n=406). In 172 scans there was a coronary bypass, in 222 scans there were missing calcified regions, in 137 scans other structures were incorrectly included in CAC segmentation, and in 343 scans there were excessive motion artifacts.

Vertebrae

For the vertebral measurements outliers to check were identified by three methods. The first method looked at the largest absolute differences in inter-vertebra height measurements in pairs of adjacent vertebra. Based on the first method, 1033 scans with the highest inter-vertebra height differences were selected for a visual check. This number was arrived at by including new scans to check until few additional errors were found. The second method looked at the intra-vertebra differences between the height measurement at the posterior/medial/anterior position of a vertebra and the leave-one-out mean of the same vertebra's posterior/medial/anterior vertebra height measurements across all of a subject's scans. All scans for the 252 subjects with the highest intra-vertebra differences were selected for a check, comprising 1004 scans. The third

method looked at the highest and lowest 1% of vertebra measurements at baseline. This corresponded to 65 scans which were not already selected for inspection by the first two methods. In total, the vertebral results were checked on 2102 scans.

The visual checks were made on the sagittal vertebra result representation provided by the Siemens AI Rad Companion Chest CT software, which showed the vertebral column with an overlay of the measurements. This view provided a convenient way to inspect the measurements. When in doubt (e.g. when the sagittal result overlay view was unclear) the full CT scan was inspected slice-by-slice to arrive at a conclusion. Individual vertebra measurements were excluded from the dataset if the measurement was deemed incorrect. If most measurements were incorrect, the entire result was considered unusable, and all the vertebra measurements were excluded. Additionally, other factors such as spinal implants or severe imaging artefacts were treated as unusable results, and all the vertebra measurements were excluded.

Out of the 2102 checked scans, a total of 118 scans were completely excluded, and 899 scans had either one or more vertebra measurement(s) excluded.

Emphysema

For emphysema, outliers were identified by looking at the highest differences of all of a subject's Low Attenuation Area Percentage (LAA%, percentage of lung voxels below -950HU) measurements across all of a subject's scans with the leave-one-out mean of the LAA% measurements of that subject. The assumption was that excessively large changes on only one scan can only be the result of an algorithm failure or inconsistent imaging factors and will not have been caused by physiological changes. The total LAA% value was chosen for the algorithm error check, because this score is dependent on both the emphysema volume segmentation as well as the total lung volume segmentation. The 200 subjects with the highest absolute LAA% value deviation (from the leave-one-out mean of all of their scans) were selected for visual check of the emphysema segmentations.

For each scan, the AI-Rad Companion results were assessed to be either sufficiently accurate, or inaccurate and unusable. The result was deemed inaccurate in the following scenarios:

- The emphysema segmentation did not correspond with emphysema on the original CT images
- The amount of emphysema was greatly overestimated.
- The amount of emphysema was greatly underestimated.
- Large parts of the lung lobe segmentation were incorrect.
- The field of view of either the original images or the result images was truncated, causing large parts of the lungs to not be analyzed.
- Results with a value of -1, indicating a complete algorithm failure (applied to the entire dataset, not only the selected 200 subjects).

The manual check was performed by viewing the CT scan in the lung setting and the emphysema segmentation overlay side-by-side and slice-by-slice. First, the scan with the most deviating result was checked, along with an adjacent follow-up result. If this did not yield a clear conclusion about which result should be marked for exclusion, then the remaining scans would also be examined.

A total of 766 scans were selected for the error check. Of these, 449 were found to be inaccurate and were excluded. In addition, all results with a LAA% value of -1 were marked as an algorithm failure, marking an additional 562 scans to be excluded. In total, 1011 scans were excluded.

Table A1: Number of scans which had at least one or all measurements excluded per screening round, based on the checked scans.

**Exclusion type**	**Round one (n=8441)**	**Round two (n=7100)**	**Round three (n=6218)**	**Round four (n=4061)**
At least one Aorta position	26	22	14	4
All Aorta positions	3	0	2	0
CACV	199	234	270	245
At least one vertebra	256	205	272	166
All vertebrae	42	22	30	24
Emphysema	397	243	201	170

For the purposes of this table short-term follow-up scans for indeterminate lung nodules are included in the screening round during which they were acquired. Number of scans in each screening round is indicated by n.

Appendix C: Example images

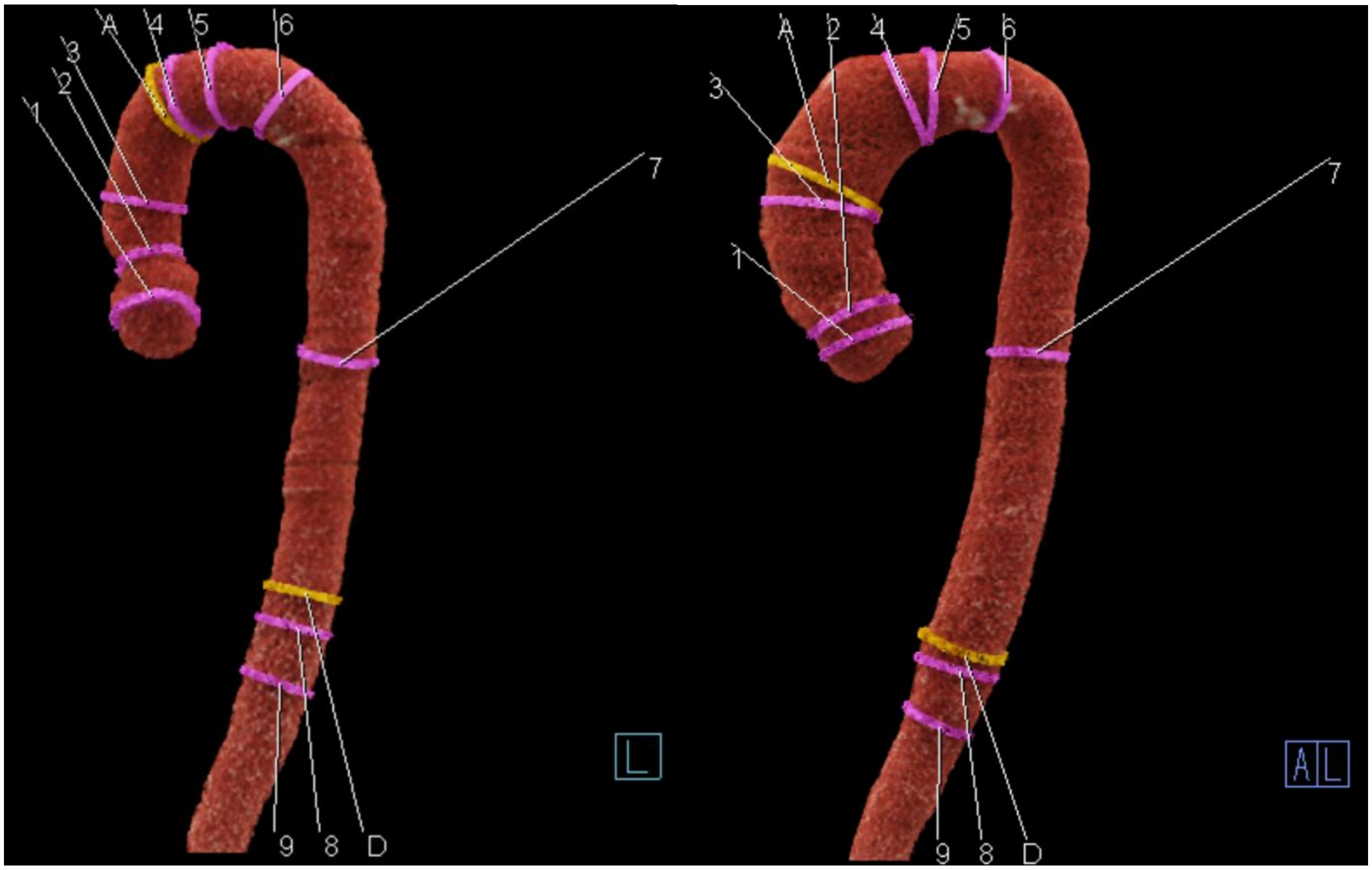

Left: all measurements correctly positioned. Right: Mid aortic arch measurement incorrectly angulated. 1: Sinus of Valsalva, 2: Sinotubular junction, 3: Mid ascending aorta, 4: Proximal Aortic arch, 5: Mid aortic arch, 6: Proximal descending aorta, 7: Mid ascending aorta, 8: Aorta at diaphragm, 9: Aorta at celiac artery origin, A: Maximum ascending aorta diameter, D: Maximum descending aorta diameter.

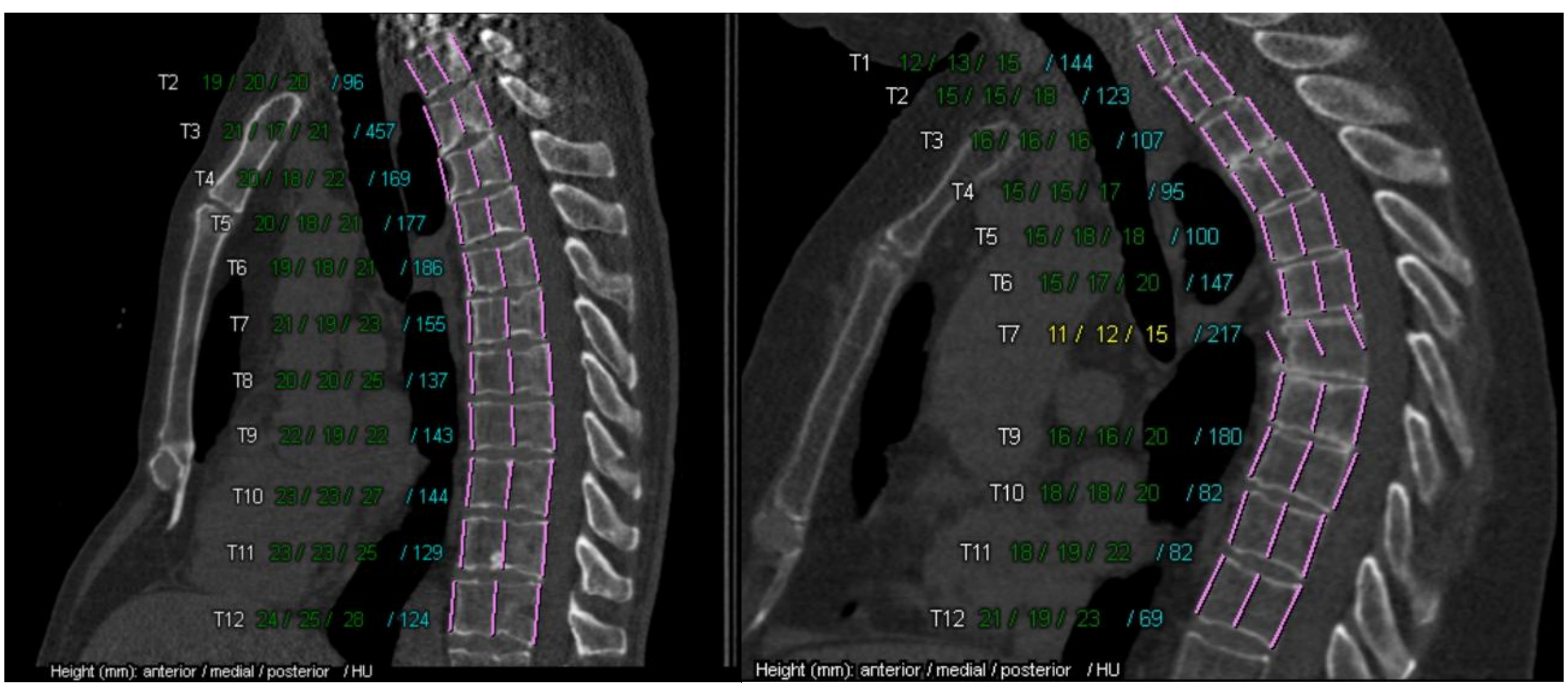

Left: all vertebrae correctly segmented. Right: Incorrect measurements of T1 due to scan noise.

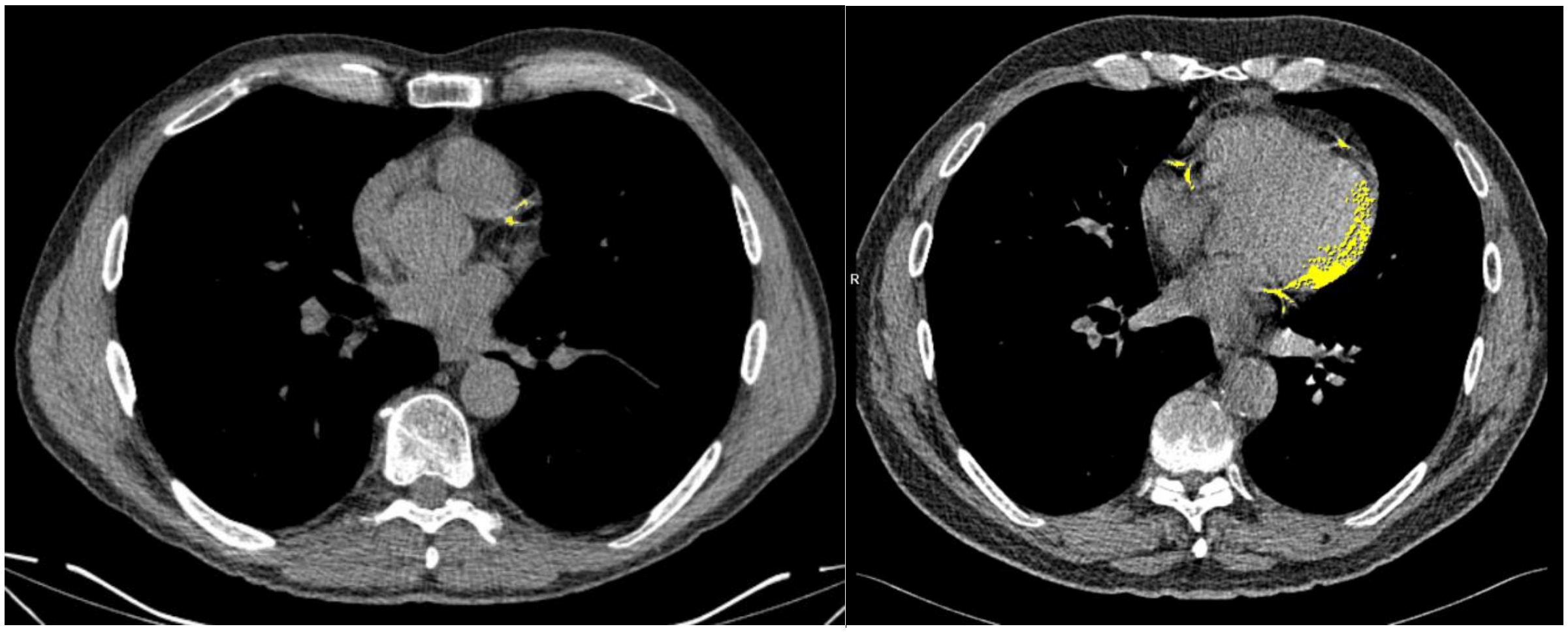

Left: coronary calcium correctly segmented. Right: excessive segmentation of coronary calcium and artefacts. Coronary calcium as detected/segmented by software displayed in yellow.

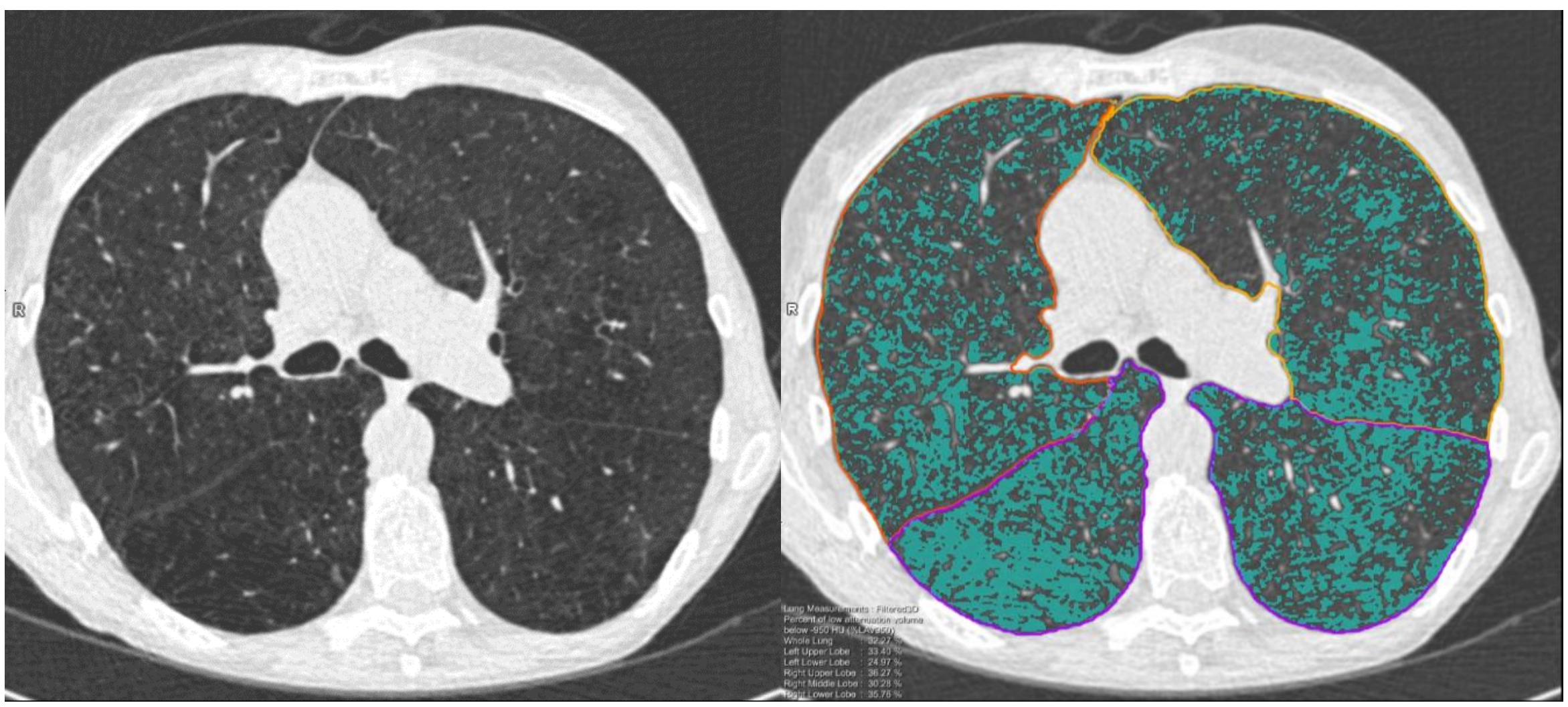

A: Left: CT slice with extensive emphysema. Right: Correct emphysema segmentation. Emphysema displayed in cyan. Coloured lines indicate outlines of lung lobes.

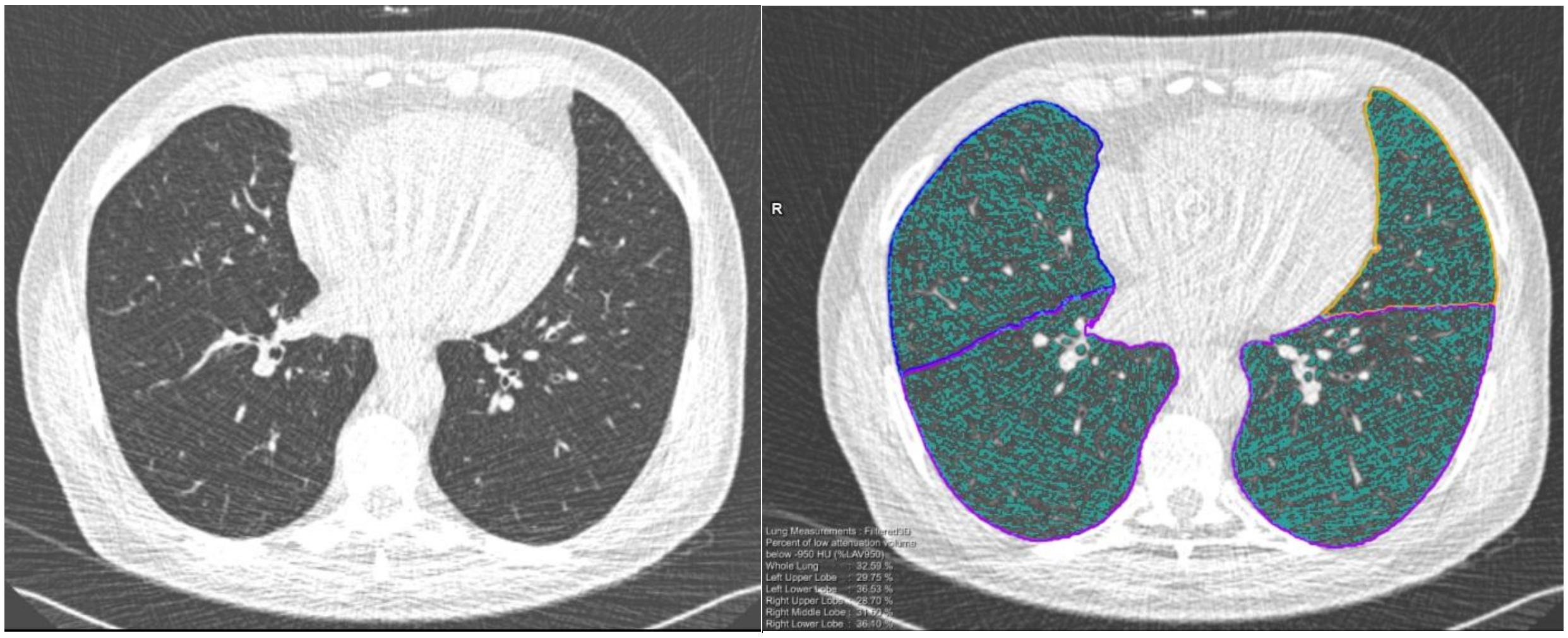

B: Left: CT slice without discernible emphysema. Right: high amounts of scan noise leading to overestimation of emphysema. Emphysema displayed in cyan. Coloured lines indicate outlines of lung lobes.

Appendix D: Full vertebrae details

Table D1. Details on vertebrae T2 through T11.

Biomarker	Baseline mean	Repeat mean	Absolute difference	Relative difference (%)	ICC	ICC 95%CI
T2 Height Anterior mm (n=1368)	18.4 ± 1.6	18.4 ± 1.6	1.1 ± 0.9	5.8 ± 5.2	0.594	(95%CI: 0.558-0.627)
T2 Height Medial mm (n=1368)	17.5 ± 1.4	17.5 ± 1.4	0.7 ± 0.7	4.0 ± 4.2	0.747	(95%CI: 0.723-0.770)
T2 Height Posterior mm (n=1368)	19.0 ± 1.6	19.1 ± 1.6	1.0 ± 1.0	5.3 ± 5.4	0.609	(95%CI: 0.575-0.641)
T2 Radiodensity HU (n=1368)	188.2 ± 46.9	188.1 ± 45.9	16.3 ± 16.4	9.0 ± 9.2	0.876	(95%CI: 0.863-0.888)
T3 Height Anterior mm (n=1406)	18.9 ± 1.5	18.8 ± 1.5	0.9 ± 0.8	5.0 ± 4.6	0.660	(95%CI: 0.629-0.688)
T3 Height Medial mm (n=1406)	17.6 ± 1.4	17.6 ± 1.4	0.7 ± 0.7	3.7 ± 4.0	0.767	(95%CI: 0.744-0.787)
T3 Height Posterior mm (n=1406)	19.6 ± 1.5	19.6 ± 1.5	0.9 ± 0.8	4.4 ± 4.1	0.680	(95%CI: 0.651-0.707)
T3 Radiodensity HU (n=1406)	186.2 ± 47.3	185.9 ± 47.2	15.1 ± 13.5	8.4 ± 7.8	0.908	(95%CI: 0.899-0.917)
T4 Height Anterior mm (n=1409)	19.3 ± 1.6	19.2 ± 1.6	0.9 ± 0.9	4.6 ± 4.6	0.689	(95%CI: 0.661-0.716)
T4 Height Medial mm (n=1409)	18.1 ± 1.4	18.1 ± 1.4	0.7 ± 0.7	3.7 ± 4.0	0.772	(95%CI: 0.750-0.792)
T4 Height Posterior mm (n=1409)	20.4 ± 1.5	20.4 ± 1.5	0.8 ± 0.8	4.1 ± 3.9	0.723	(95%CI: 0.697-0.747)

T4 Radiodensity HU (n=1409)	173.9 ± 43.6	173.0 ± 42.5	13.6 ± 13.4	8.1 ± 7.6	0.902	(95%CI: 0.892-0.911)
T5 Height Anterior mm (n=1408)	19.6 ± 1.6	19.6 ± 1.7	0.9 ± 0.9	4.5 ± 4.5	0.723	(95%CI: 0.697-0.747)
T5 Height Medial mm (n=1408)	18.6 ± 1.4	18.6 ± 1.5	0.6 ± 0.7	3.5 ± 3.9	0.781	(95%CI: 0.760-0.801)
T5 Height Posterior mm (n=1408)	21.2 ± 1.6	21.2 ± 1.6	0.8 ± 0.8	3.9 ± 3.9	0.740	(95%CI: 0.716-0.763)
T5 Radiodensity HU (n=1408)	167.9 ± 41.5	167.0 ± 41.5	12.1 ± 12.5	7.5 ± 7.4	0.912	(95%CI: 0.903-0.921)
T6 Height Anterior mm (n=1410)	19.6 ± 1.7	19.6 ± 1.7	0.8 ± 0.9	4.2 ± 4.7	0.745	(95%CI: 0.721-0.767)
T6 Height Medial mm (n=1410)	18.9 ± 1.5	18.9 ± 1.5	0.6 ± 0.8	3.4 ± 5.1	0.762	(95%CI: 0.739-0.783)
T6 Height Posterior mm (n=1410)	21.9 ± 1.7	21.9 ± 1.7	0.9 ± 0.8	3.9 ± 3.7	0.754	(95%CI: 0.731-0.776)
T6 Radiodensity HU (n=1410)	160.9 ± 41.5	159.6 ± 40.9	12.4 ± 13.7	8.0 ± 8.3	0.900	(95%CI: 0.889-0.909)
T7 Height Anterior mm (n=1408)	19.5 ± 1.8	19.5 ± 1.7	0.8 ± 0.9	4.3 ± 5.2	0.766	(95%CI: 0.743-0.787)
T7 Height Medial mm (n=1408)	19.0 ± 1.6	19.0 ± 1.6	0.6 ± 0.7	3.4 ± 4.0	0.806	(95%CI: 0.787-0.824)
T7 Height Posterior mm (n=1408)	22.3 ± 1.7	22.3 ± 1.7	0.8 ± 0.8	3.8 ± 3.7	0.770	(95%CI: 0.747-0.790)
T7 Radiodensity HU (n=1408)	153.2 ± 42.6	152.8 ± 42.6	12.4 ± 12.8	8.5 ± 8.6	0.913	(95%CI: 0.904-0.921)
T8 Height Anterior mm (n=1409)	19.8 ± 1.8	19.8 ± 1.8	0.8 ± 0.8	4.1 ± 4.3	0.803	(95%CI: 0.783-0.820)
T8 Height Medial mm (n=1409)	19.3 ± 1.7	19.3 ± 1.6	0.7 ± 0.7	3.5 ± 4.4	0.819	(95%CI: 0.801-0.835)
T8 Height Posterior mm (n=1409)	22.6 ± 1.7	22.6 ± 1.7	0.8 ± 0.8	3.6 ± 3.5	0.786	(95%CI: 0.765-0.805)
T8 Radiodensity HU (n=1409)	145.3 ± 41.9	144.9 ± 41.9	12.3 ± 13.7	9.4 ± 16.7	0.904	(95%CI: 0.894-0.913)
T9 Height Anterior mm (n=1406)	20.7 ± 1.9	20.7 ± 1.9	0.9 ± 0.9	4.4 ± 4.5	0.772	(95%CI: 0.751-0.793)
T9 Height Medial mm (n=1406)	20.0 ± 1.7	20.0 ± 1.7	0.7 ± 0.8	3.6 ± 3.9	0.804	(95%CI: 0.785-0.822)
T9 Height Posterior mm (n=1406)	23.1 ± 1.7	23.1 ± 1.7	0.8 ± 0.8	3.7 ± 3.5	0.766	(95%CI: 0.744-0.787)
T9 Radiodensity HU (n=1406)	147.1 ± 42.4	146.2 ± 41.9	12.4 ± 14.0	8.9 ± 10.2	0.902	(95%CI: 0.892-0.911)
T10 Height Anterior mm (n=1405)	22.3 ± 1.9	22.2 ± 1.9	1.0 ± 1.0	4.4 ± 4.6	0.744	(95%CI: 0.719-0.766)
T10 Height Medial mm (n=1405)	21.5 ± 1.7	21.5 ± 1.8	0.8 ± 0.9	3.7 ± 4.1	0.780	(95%CI: 0.759-0.800)

T10 Height Posterior mm (n=1405)	24.3 ± 1.7	24.3 ± 1.7	0.9 ± 0.8	3.6 ± 3.5	0.759	(95%CI: 0.736-0.780)
T10 Radiodensity HU (n=1405)	153.5 ± 43.2	151.6 ± 42.4	12.1 ± 15.1	8.3 ± 10.0	0.898	(95%CI: 0.887-0.908)
T11 Height Anterior mm (n=1381)	23.0 ± 2.0	23.0 ± 2.0	1.0 ± 0.9	4.3 ± 4.1	0.765	(95%CI: 0.743-0.786)
T11 Height Medial mm (n=1381)	23.0 ± 1.8	23.0 ± 1.8	0.8 ± 0.8	3.4 ± 3.8	0.801	(95%CI: 0.782-0.820)
T11 Height Posterior mm (n=1381)	26.3 ± 1.8	26.2 ± 1.9	0.9 ± 0.9	3.6 ± 3.7	0.747	(95%CI: 0.723-0.769)
T11 Radiodensity HU (n=1381)	143.8 ± 40.0	142.9 ± 39.5	12.2 ± 13.1	9.0 ± 10.0	0.899	(95%CI: 0.889-0.909)

Values are reported as mean ± standard deviation. HU: Hounsfield Unit. T2: Second Thoracic Vertebrae, etc.

Appendix E: Vertebral height loss analysis

Vertebral height loss calculation

For the derived measurement of vertebral height loss, we used the Genant semi-quantitative method, where we compared anterior, medial and posterior heights to the heights of the vertebra below or above and expressed these height ratios as a percentage of height loss.> This yielded one vertebral height loss percentage per vertebra per scan. Per scan, only the measurement for the vertebra with the highest height loss percentage (most height loss) was used in the analysis.[2]

Vertebral height loss percentage was divided into four categories; none, mild, moderate, and severe. It was defined as none: <20%, mild: ≥20-<25%, moderate: ≥25-<40%, and severe: ≥40%.[1,>]

Vertebral height loss results

Vertebra height loss percentage had a mean relative difference of 20.3%. Only moderate agreement (0.5< ICC < 0.75) was observed for the vertebral height loss percentage measurement, with an ICC of 0.540 (95%CI: 0.502-0.576). Vertebral height loss percentage presence and severity categorization had Cohen's kappa's of 0.40 and 0.41, respectively. The presence kappa was unweighted and based on two groups; none/mild and moderate/severe. The Severity kappa was quadratically weighted and based on the four individual severity categories of none, mild, moderate, and severe. Moderate or severe vertebral height loss (loss >25%) were present in 5.6% of 1420 subjects.

Compared to the findings for the incidental findings in the main paper, the measurement of vertebral height loss percentage and categorization into severity showed worse repeatability and agreement. It is important to note that this measurement is derived from the anterior, medial, and posterior vertebral height measurements. These underlying vertebral height measurements have excellent agreement and repeatability. Therefore, moderate agreement of height loss and poor concordance and agreement of the height loss categorization are likely the result of poor scan quality and error accumulation. It has been shown in a previous study by Buckens et al, who used the same height loss grading as this analysis, that in a subset of 575 NELSON subjects 39% had vertebral fractures, which is more than the 19.7% fracture rate we found.> They also found significant relationships between the vertebral fracture severity and all-cause mortality. NELSON scans are of comparatively poor quality due to the state of technology when they were acquired, which may indicate that human observers are more capable at accurately assessing vertebral height loss on poor quality scans. Additionally, previous research has shown that automated fracture assessment is possible using a more complex method.[3] Based on this, in combination with the poor repeatability of the vertebral height loss measurement in this analysis, one may conclude that a height loss measurement derived from anterior, medial, and posterior vertebral height measurements is insufficient, and a more complex height loss assessment method is required.

Table E1. Baseline and repeat measurement characteristics of vertebrae

Biomarker	Baseline	Repeat
Anterior height mm (n=1421)	20.1 ± 1.2	20.1 ± 1.2
Medial height mm (n=1421)	19.4 ± 1.2	19.4 ± 1.2
Posterior height mm (n=1421)	22.1 ± 1.3	22.1 ± 1.3
Radiodensity HU (n=1421)	161.9 ± 38.5	161.1 ± 38.0
Height loss % (n=1420)	16.5 ± 5.1	16.5 ± 4.9
Osteopenia % (<115HU) (n=1421)	10.3	10.5
Moderate or severe height loss % (>25%) (n=1420)	5.0	4.6

Values are reported as mean ± standard deviation, median (interquartile range), or percentage. HU: Hounsfield Unit.

Table E2. Repeatability and agreement of vertebrae measurements

Biomarker	Absolute difference	Relative difference (%)	ICC	ICC 95%CI
Vertebrae				
Anterior height mm (n=1421)	0.4 ± 0.3	1.8 ± 1.6	0.914	(95%CI: 0.905-0.922)
Medial height mm (n=1421)	0.3 ± 0.3	1.5 ± 1.4	0.948	(95%CI: 0.942-0.953)
Posterior height mm (n=1421)	0.4 ± 0.4	1.7 ± 1.6	0.927	(95%CI: 0.919-0.934)
Radiodensity HU (n=1421)	7.6 ± 9.4	4.8 ± 5.6	0.950	(95%CI: 0.945-0.955)
Height loss % (n=1420)	3.4 ± 3.1	20.3 ± 16.4	0.569	(95%CI: 0.532-0.603)

Absolute and relative difference are reported as mean ± standard deviation or median (interquartile range). HU: Hounsfield Unit.

Appendix F: Confusion matrices for categorized variables

Table F1. CACV Confusion Matrix

		Baseline					
		none	mild	moderate	severe	Total	Concordance (%)
	none	**215**	74	4	0	293	73.4
	mild	109	**293**	46	6	454	64.5
	moderate	1	50	**177**	37	265	66.8
Repeat	severe	0	7	40	**308**	355	86.8
	Total	325	424	267	351	1367	72.6

CACV: Coronary artery calcium volume. CACV categories: none: 0 mm3, mild: >0-100 mm3, moderate >100-300mm3, severe >=300 mm3.

Table F2. Emphysema LAA% Confusion Matrix

		Baseline					
		none	mild	moderate	severe	Total	Concordance (%)
	none	**710**	48	0	0	758	93.7
	mild	62	**162**	40	0	264	61.4
	moderate	10	48	**88**	0	146	60.3
Repeat	severe	1	7	18	**125**	151	82.8
	Total	783	265	146	125	1319	82.3

LAA%: Low Attenuation Area Percentage. LAA% categories: none: <5%, mild, >=5%-<10%, moderate, >=10%-<15%, and severe: >=15%.

Table F3. Maximum Ascending Aorta Diameter Confusion Matrix

		Baseline			
		<=45mm	>45mm	Total	Concordance (%)
	<=45mm	**1354**	21	1375	98.5
Repeat	>45mm	13	**39**	52	75.0
	Total	1367	60	1427	97.6

<=45mm indicates no dilatation, >45mm indicates dilatation.

Table F4. Vertebra Height Loss Percentage Confusion Matrix

		Baseline					
		none	mild	moderate	severe	Total	Concordance (%)
Repeat	none	**992**	133	25	0	1150	86.3
	mild	128	**54**	17	0	199	27.1
	moderate	25	11	**21**	0	57	36.8
	severe	0	0	4	**4**	8	50.0
	Total	1145	198	67	4	1414	75.7

Vertebral height loss percentage categories: none: <20%, mild: >=20-<25%, moderate: >=25-40%, severe: >=40%.

Table F5. Vertebral Radiodensity Confusion Matrix

		Baseline			
		<=115HU	>115HU	Total	Concordance (%)
Repeat	<=115HU	**126**	23	149	84.6
	>115HU	20	**1246**	1266	98.4
	Total	146	1269	1415	97.0

<=115HU indicates osteopenia, >115HU indicates no osteopenia.

Appendix G: Bland-Altman plots

Aorta

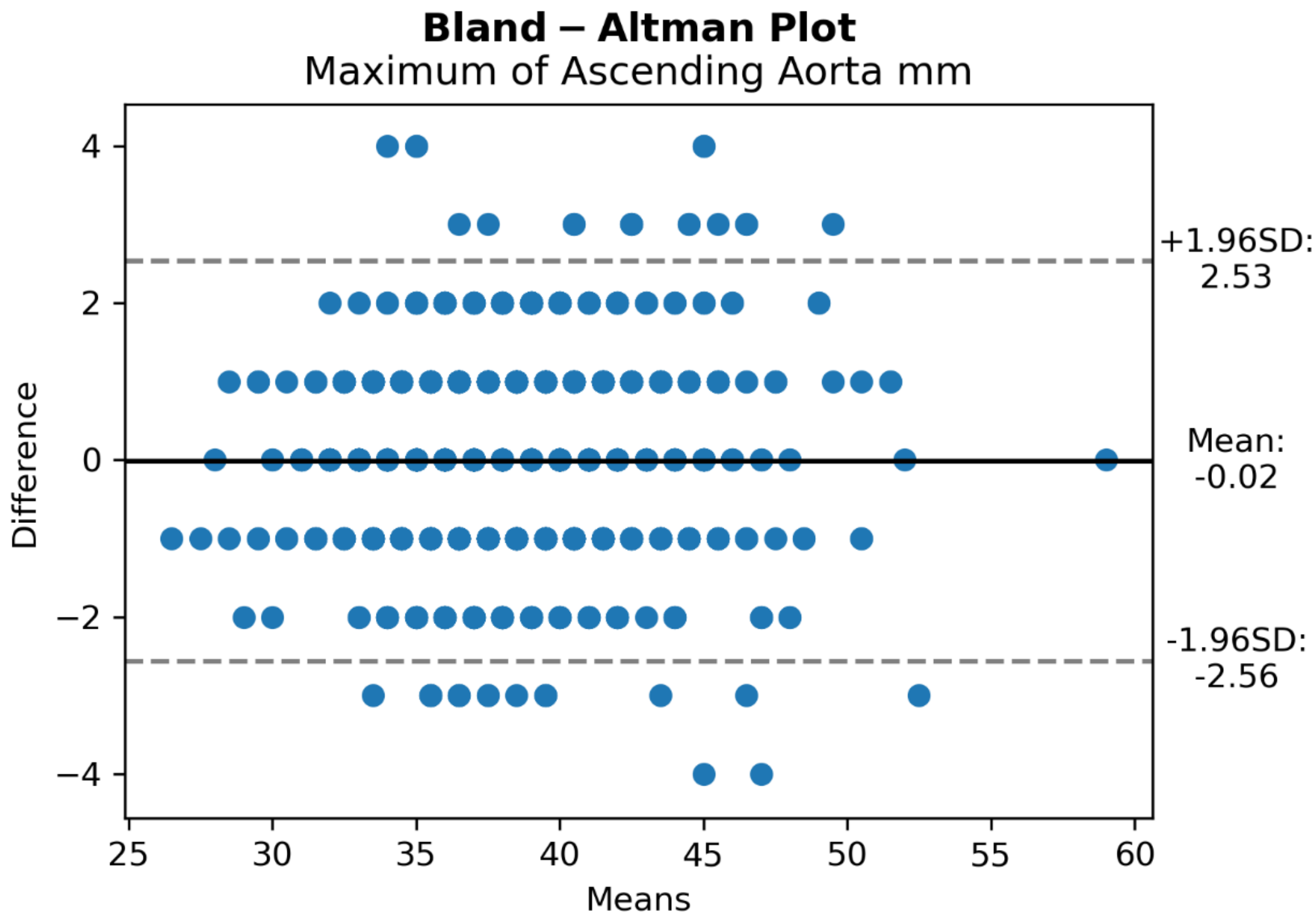

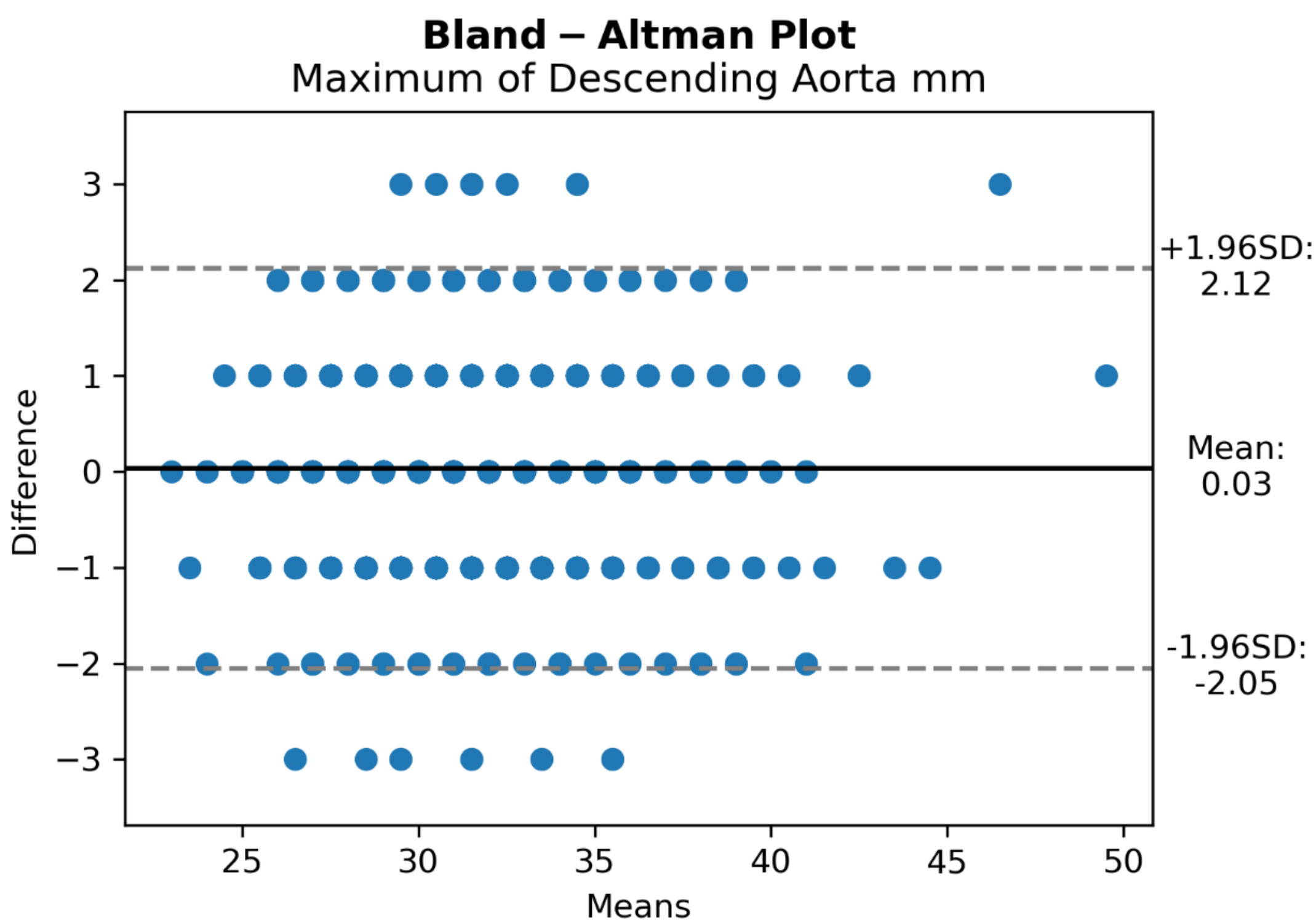

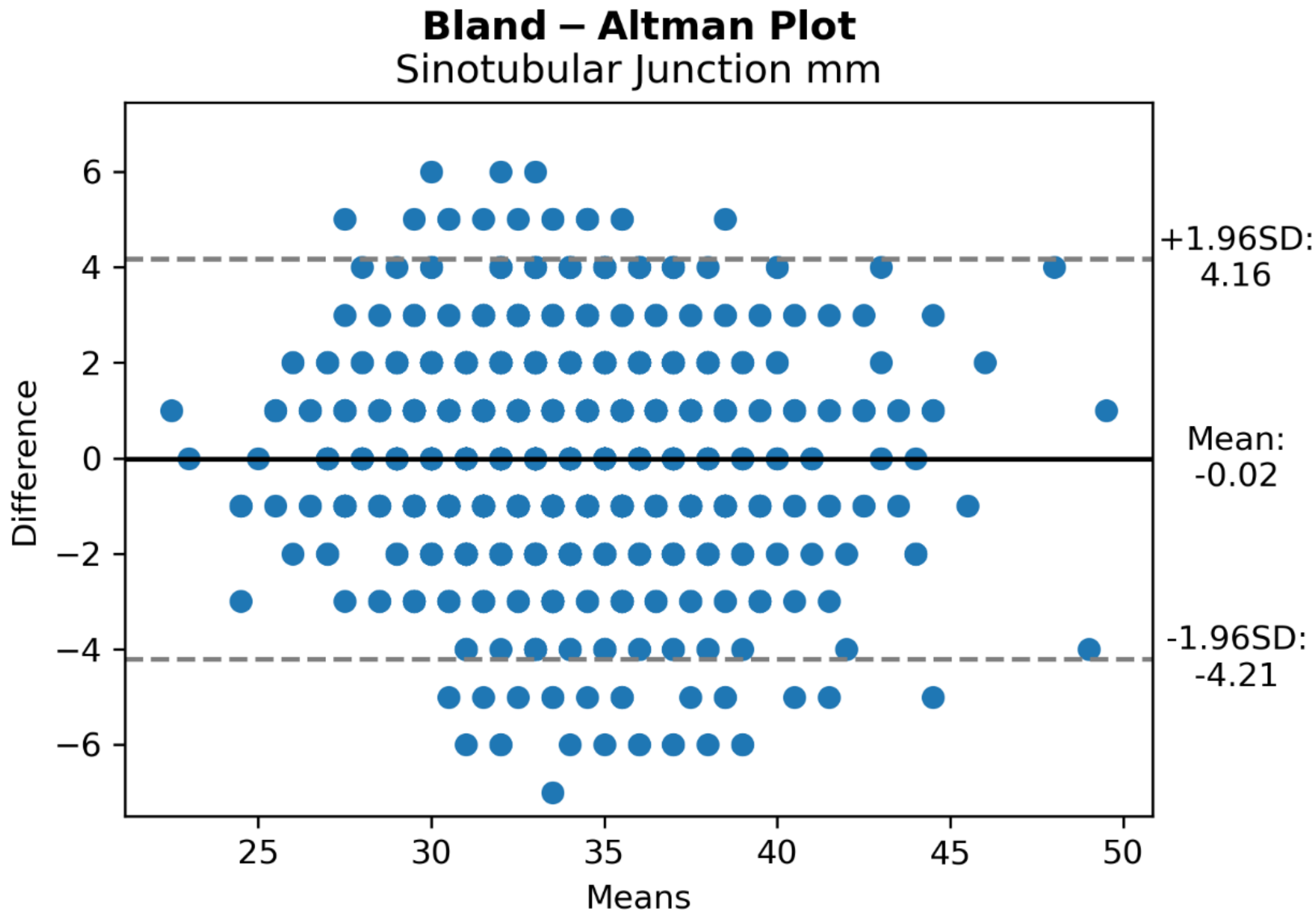
Bland – Altman Plot
Sinotubular Junction mm
6
4
2
0
−2
−4
−6
Difference
25
30
35
40
45
50
Means
+1.96SD:
4.16
Mean:
-0.02
-1.96SD:
-4.21

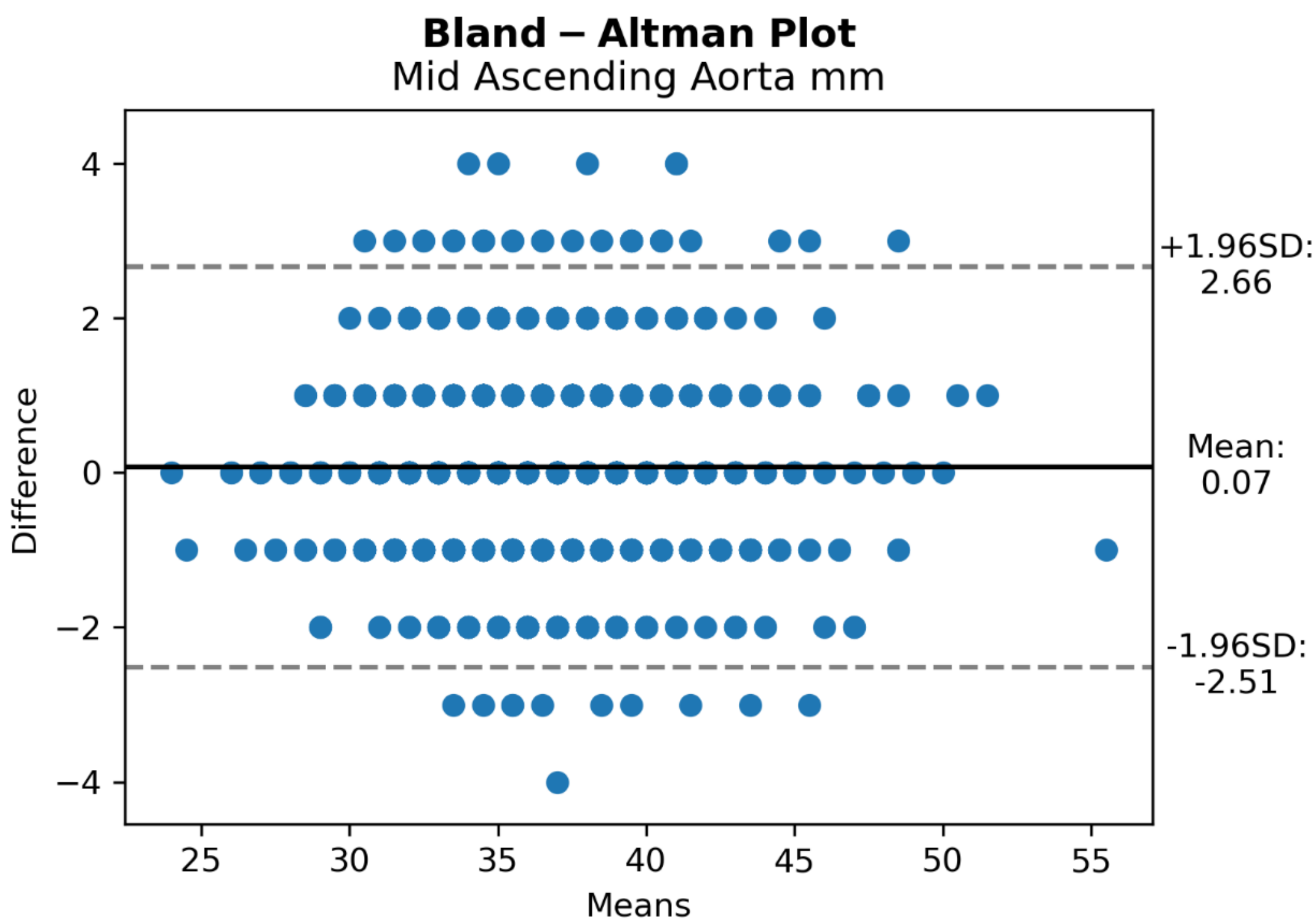
Bland – Altman Plot
Mid Ascending Aorta mm
4
2
0
−2
−4
Difference
25
30
35
40
45
50
55
Means
+1.96SD:
2.66
Mean:
0.07
-1.96SD:
-2.51

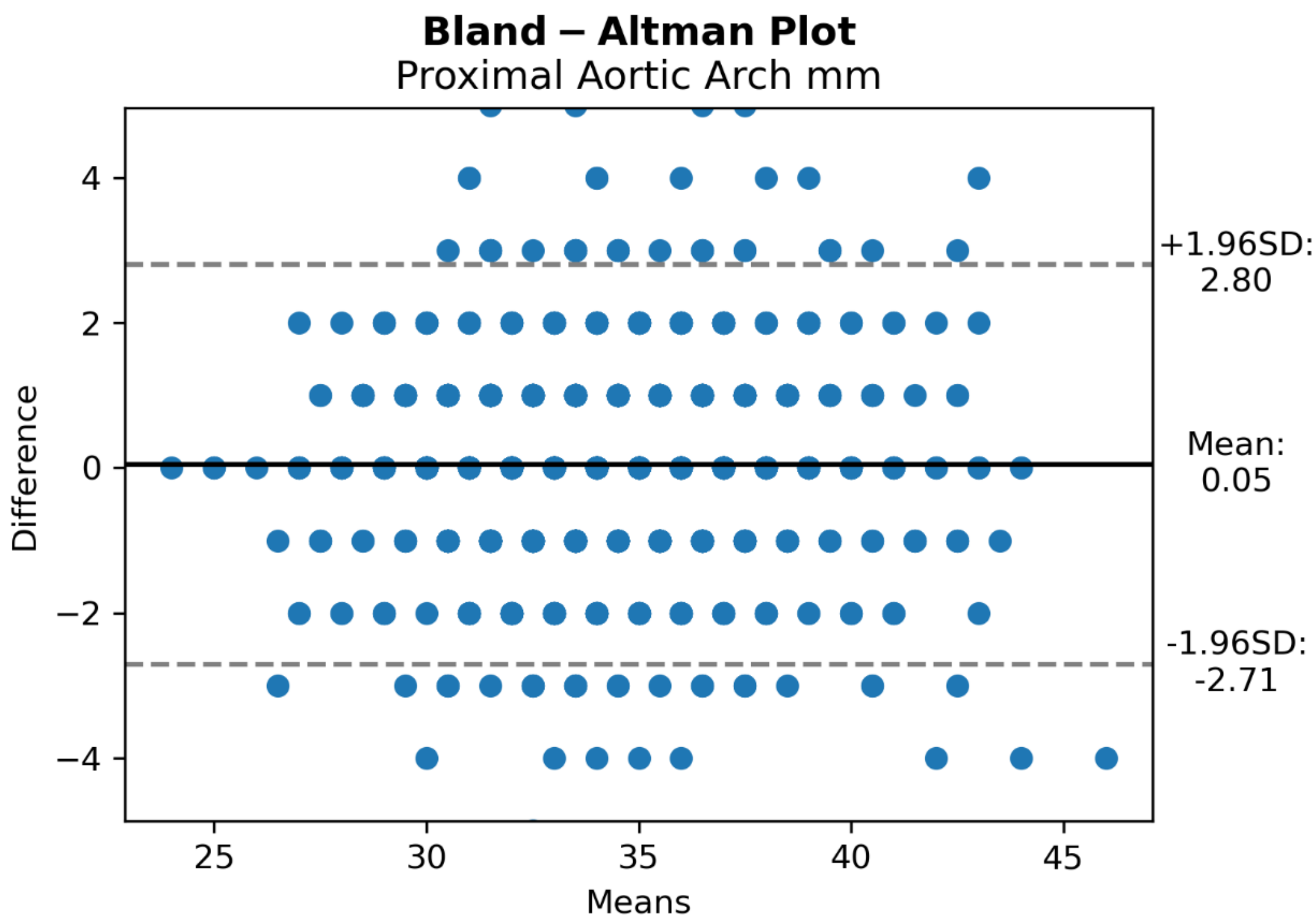

Bland – Altman Plot
Proximal Aortic Arch mm
4
2
0
−2
−4
Difference
25
30
35
40
45
Means
+1.96SD:
2.80
Mean:
0.05
-1.96SD:
-2.71

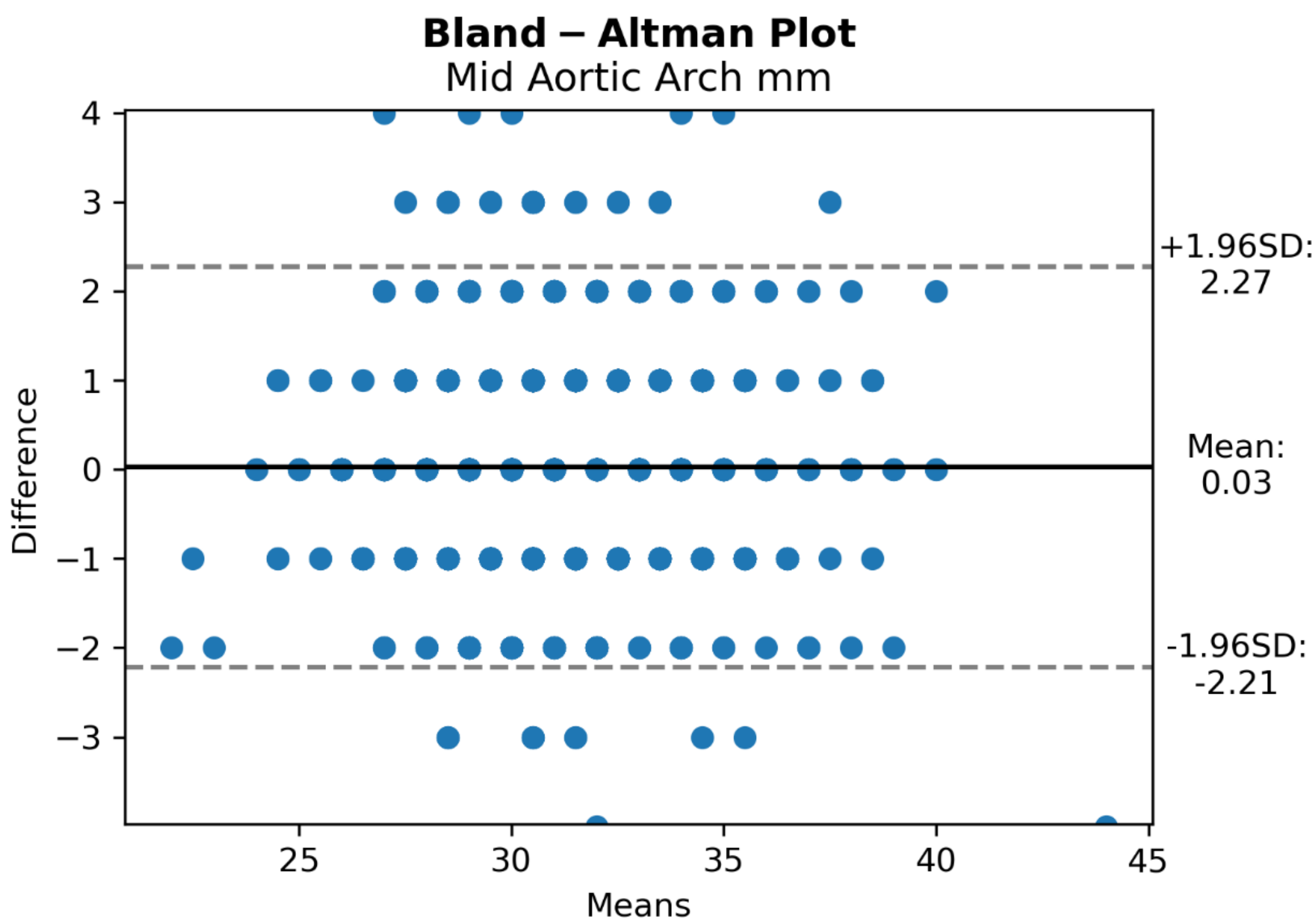

Bland – Altman Plot
Mid Aortic Arch mm
4
3
2
1
0
−1
−2
−3
Difference
25
30
35
40
45
Means
+1.96SD:
2.27
Mean:
0.03
-1.96SD:
-2.21

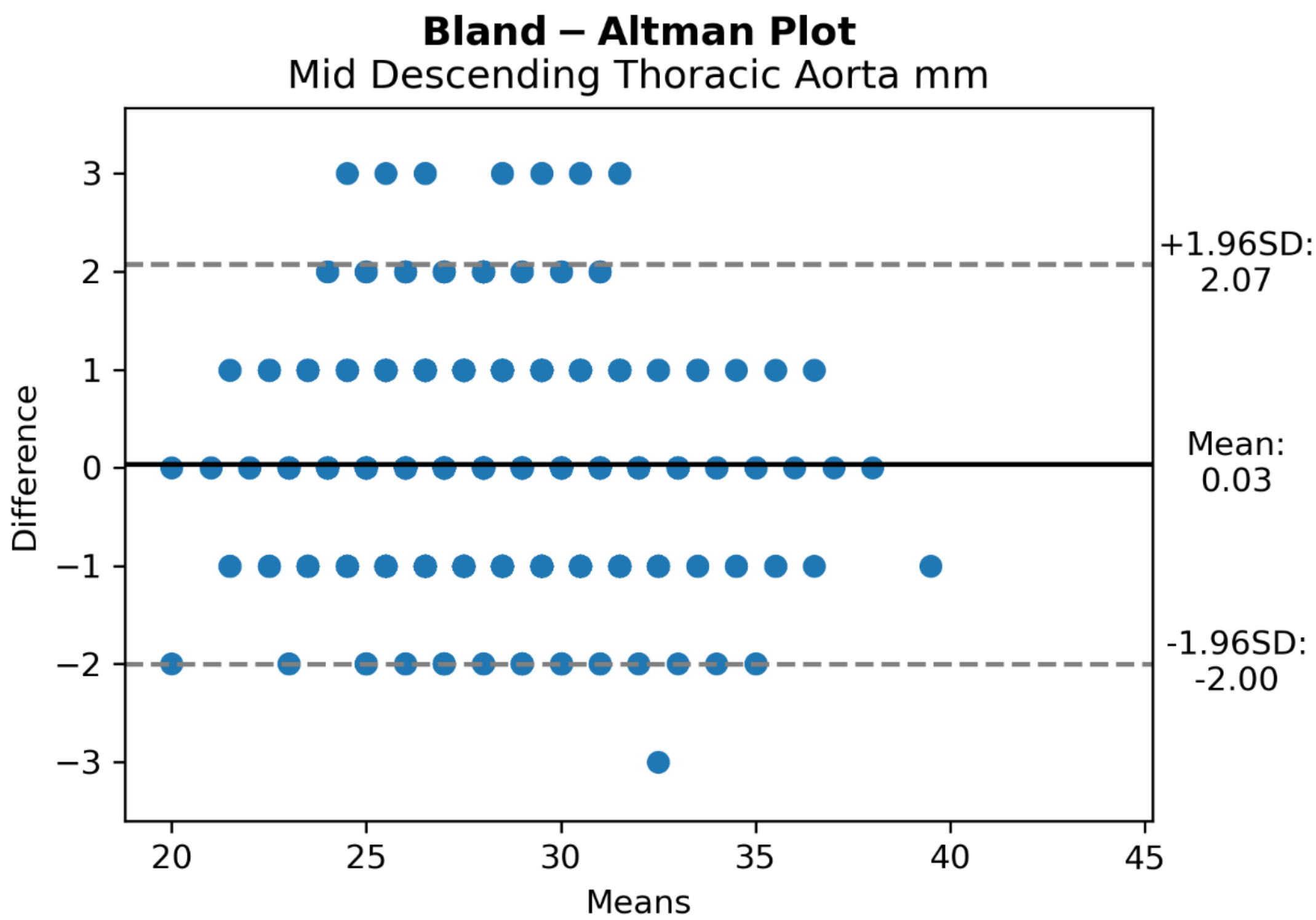
Bland – Altman Plot
Mid Descending Thoracic Aorta mm
Difference
Means
+1.96SD:
2.07
Mean:
0.03
-1.96SD:
-2.00
3
2
1
0
−1
−2
−3
20
25
30
35
40
45

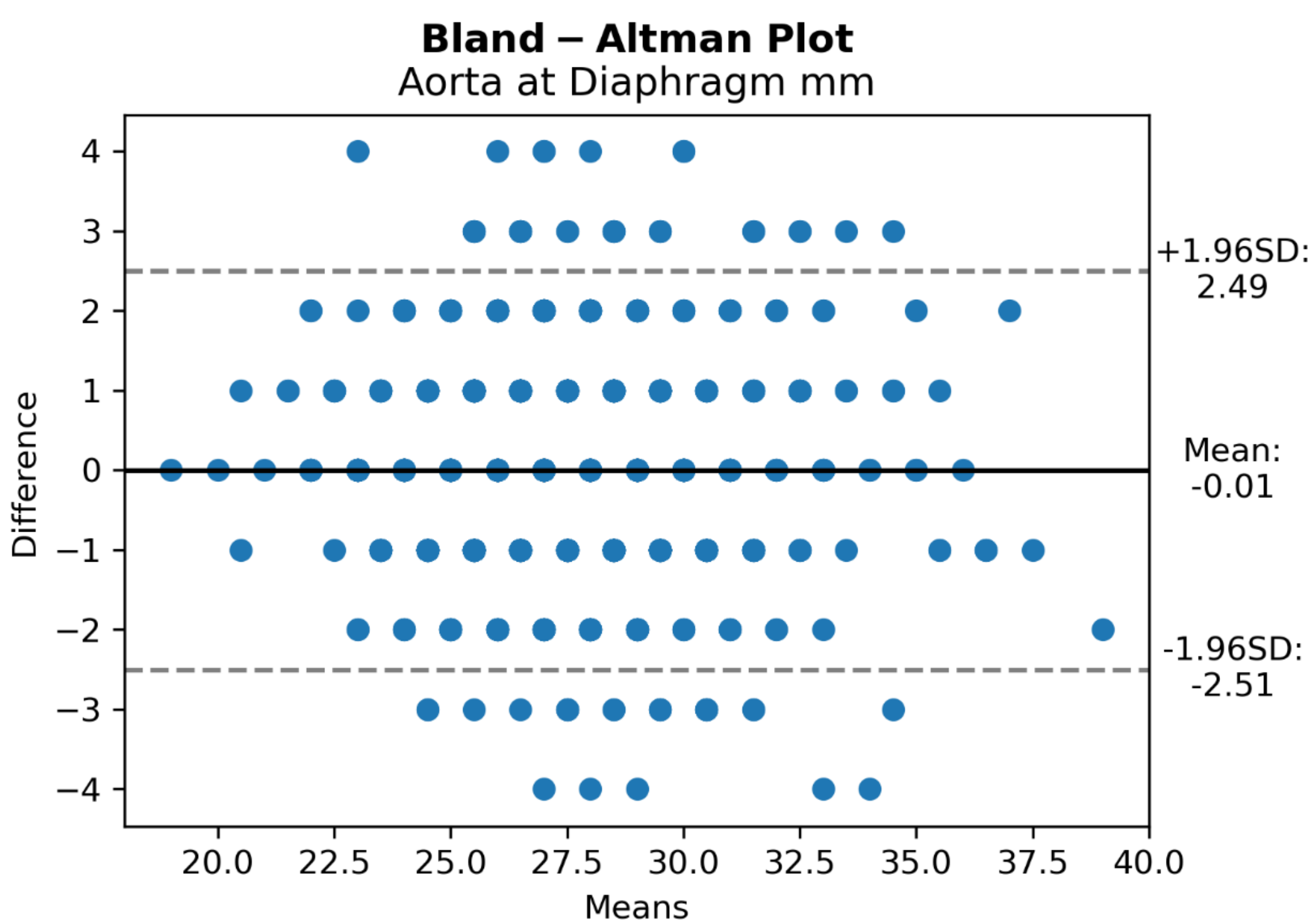
Bland – Altman Plot
Aorta at Diaphragm mm
Difference
Means
+1.96SD:
2.49
Mean:
-0.01
-1.96SD:
-2.51
4
3
2
1
0
−1
−2
−3
−4
20.0
22.5
25.0
27.5
30.0
32.5
35.0
37.5
40.0

Coronary calcium

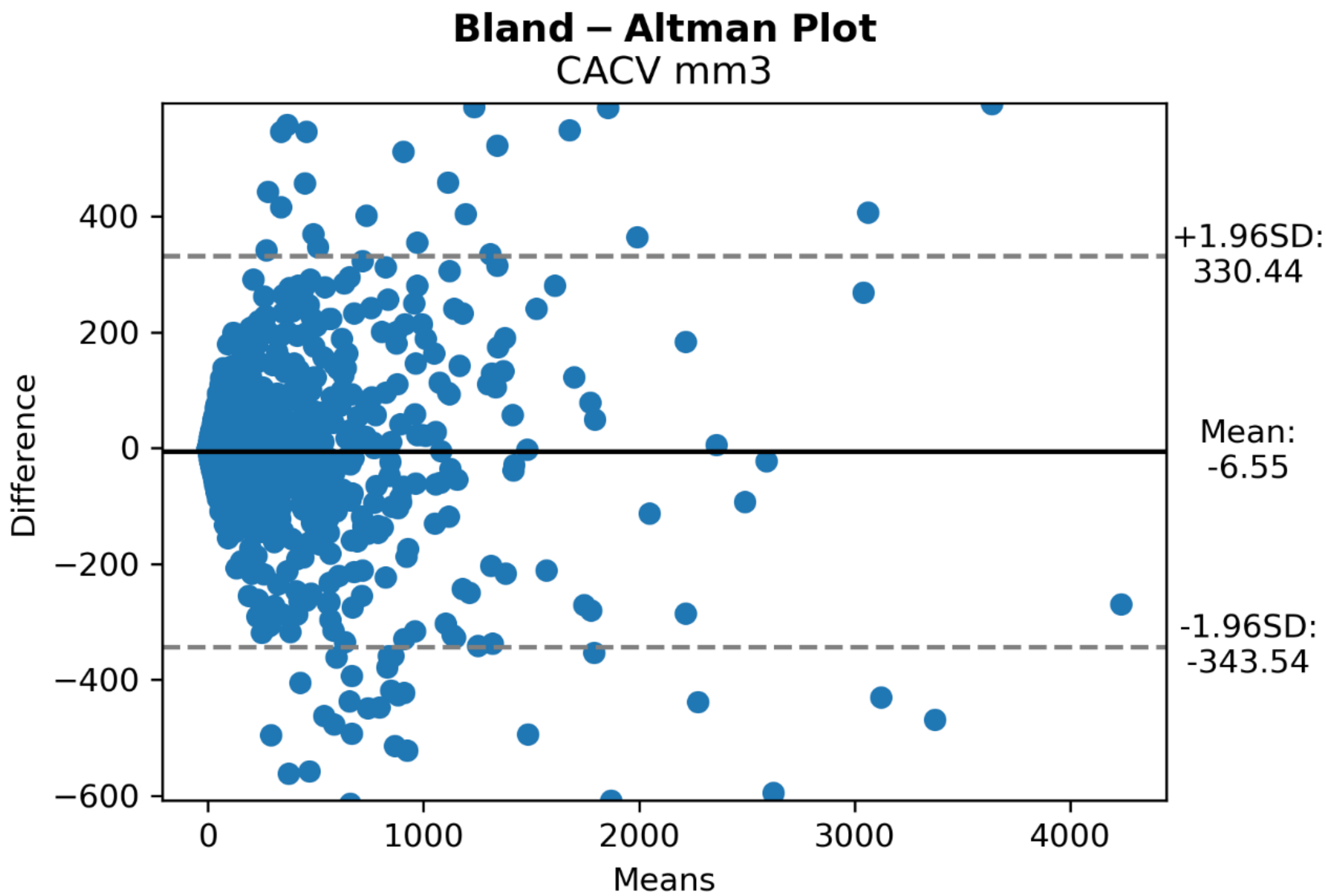

Vertebrae

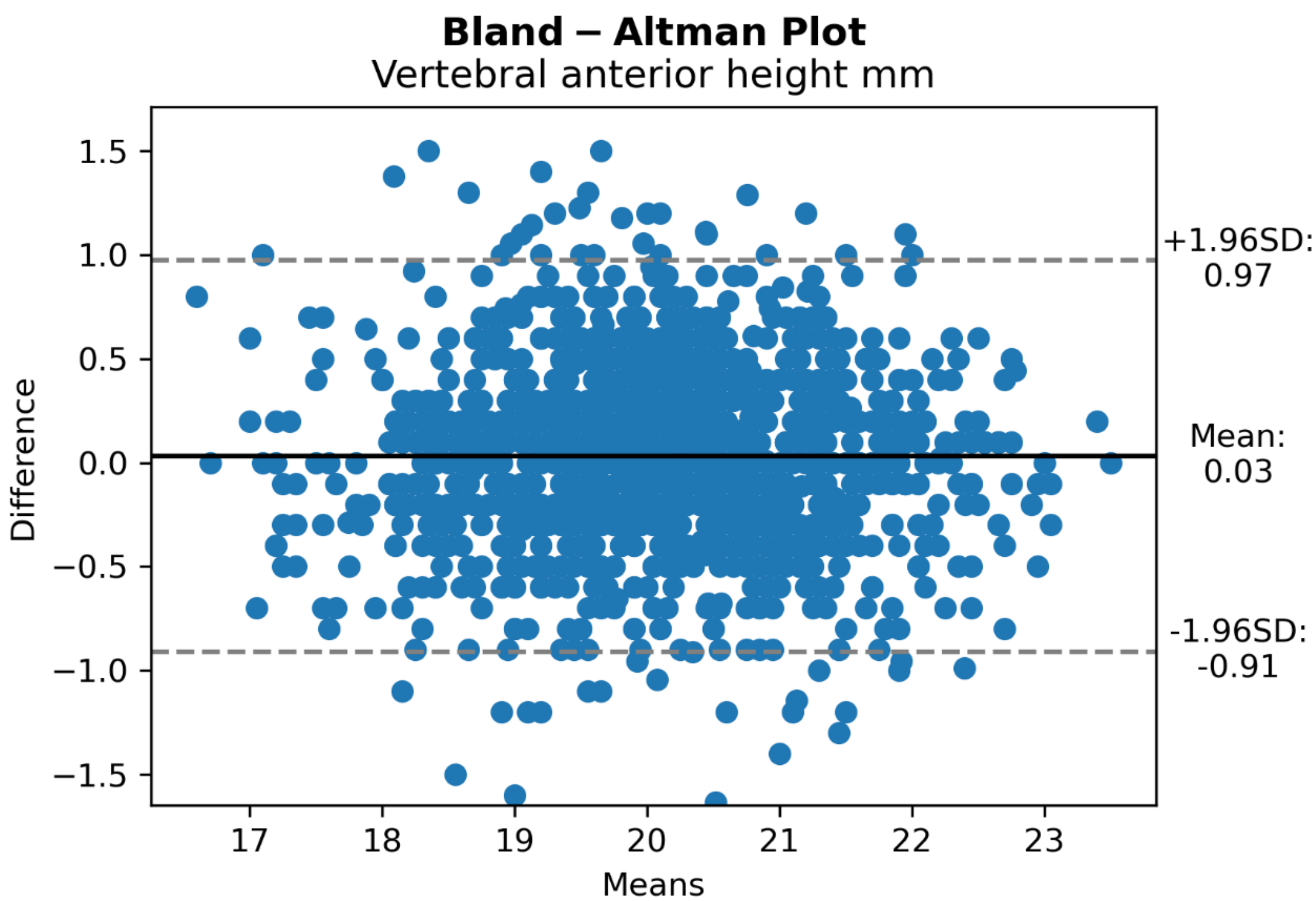

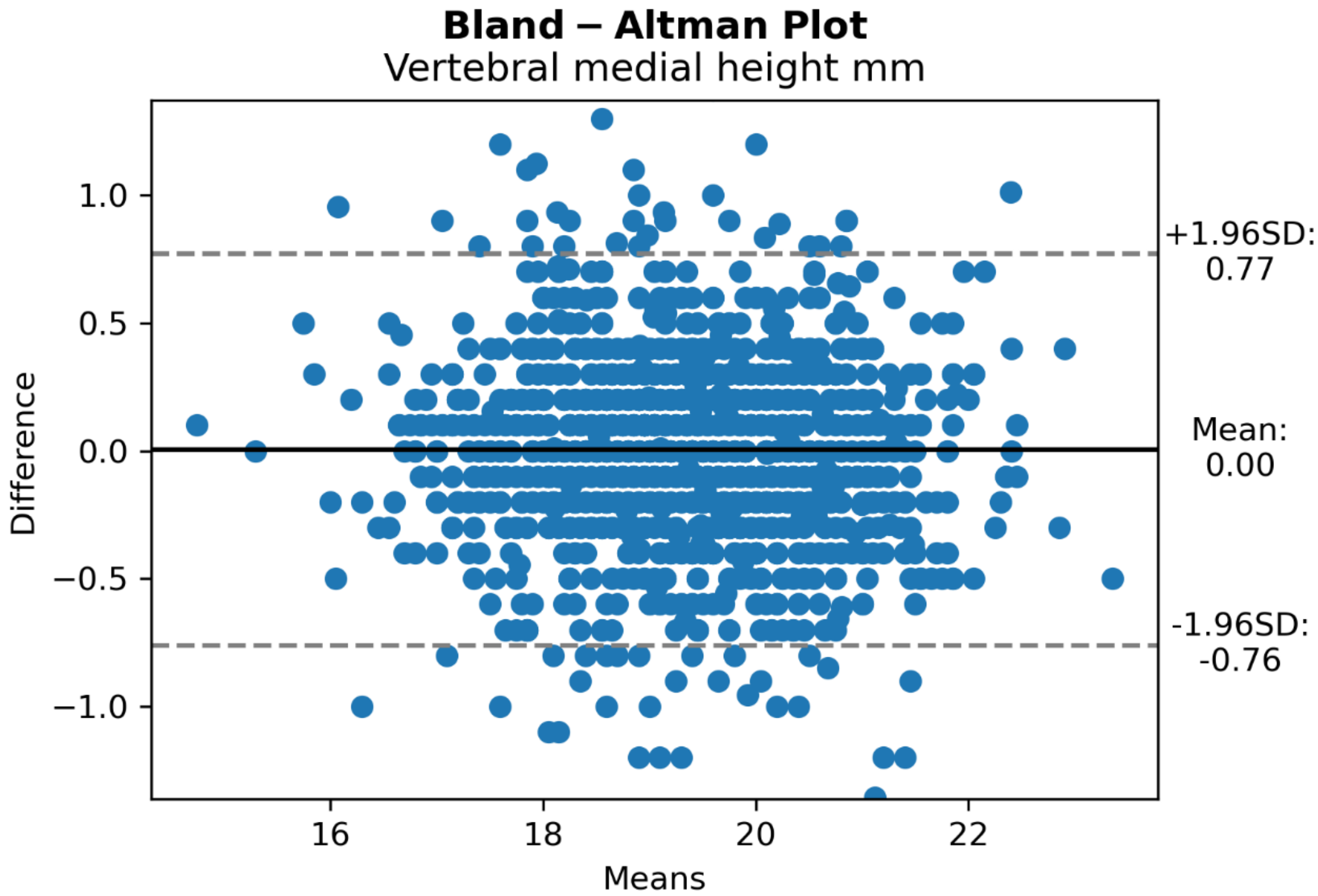
Bland – Altman Plot
Vertebral medial height mm
1.0
0.5
0.0
−0.5
−1.0
Difference
16
18
20
22
Means
+1.96SD:
0.77
Mean:
0.00
-1.96SD:
-0.76

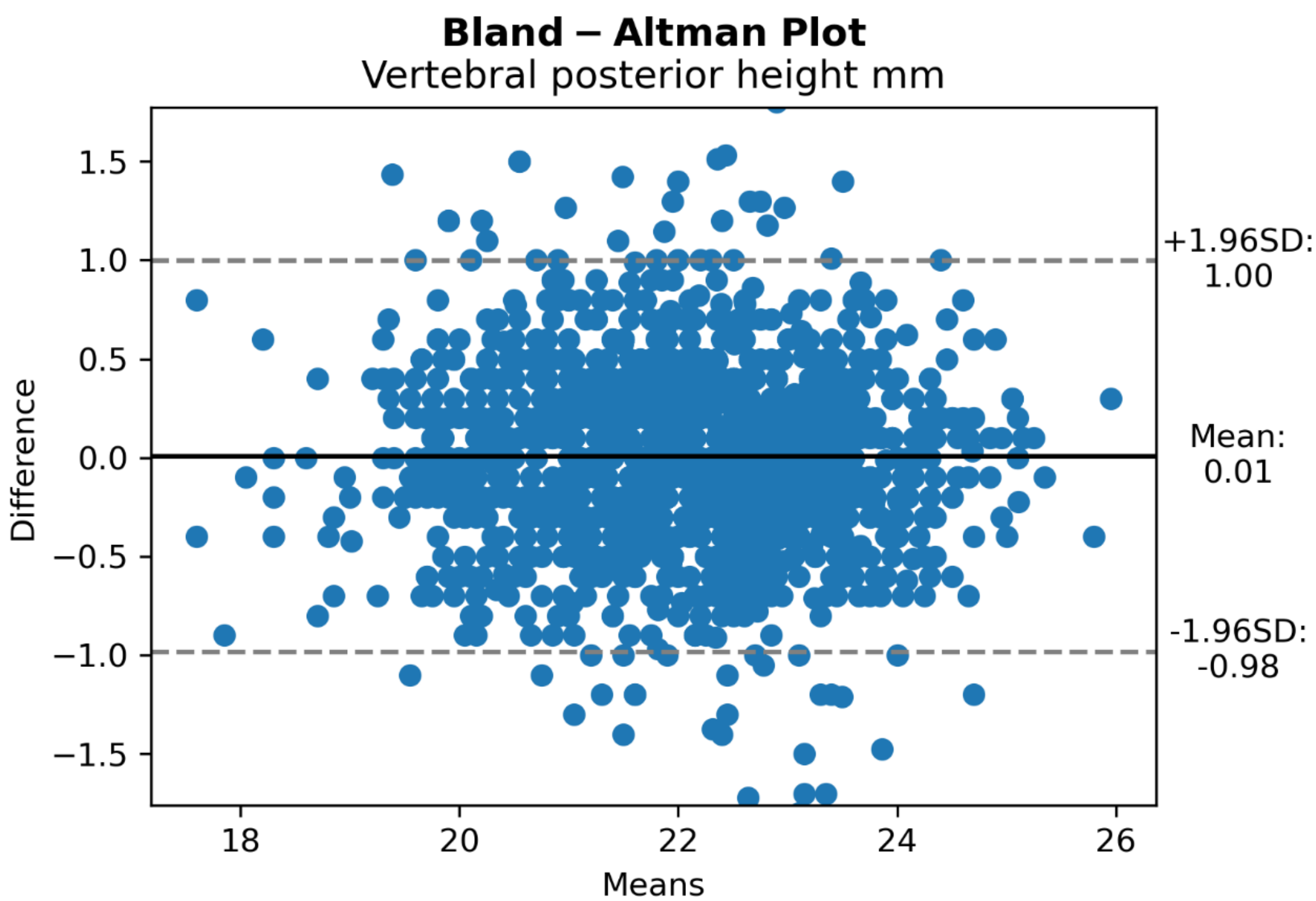
Bland – Altman Plot
Vertebral posterior height mm
1.5
1.0
0.5
0.0
−0.5
−1.0
−1.5
Difference
18
20
22
24
26
Means
+1.96SD:
1.00
Mean:
0.01
-1.96SD:
-0.98

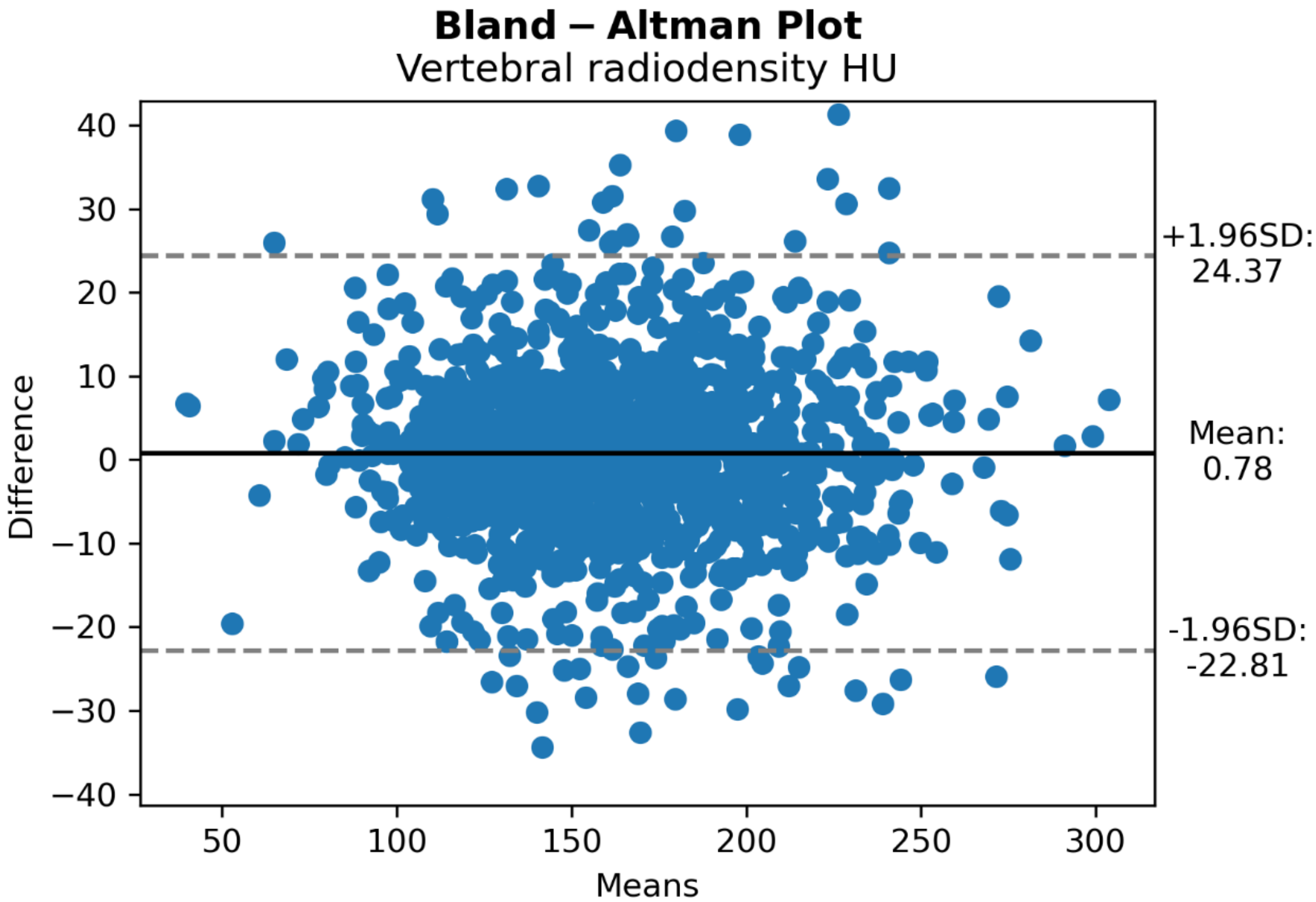
Bland – Altman Plot
Vertebral radiodensity HU
+1.96SD:
24.37
Mean:
0.78
-1.96SD:
-22.81
Difference
Means

Emphysema

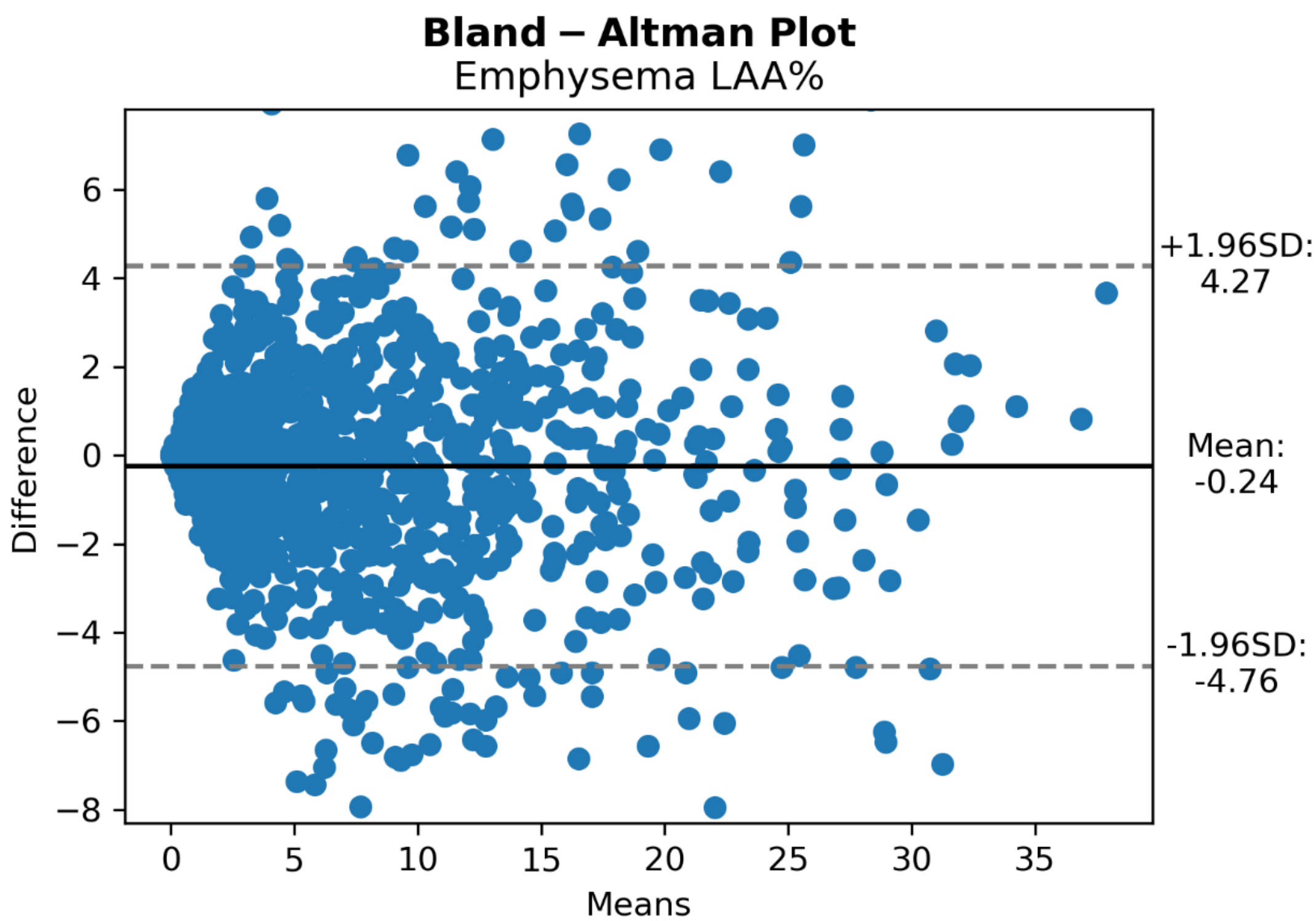
Bland – Altman Plot
Emphysema LAA%
+1.96SD:
4.27
Mean:
-0.24
-1.96SD:
-4.76
Difference
Means

Bibliography

1 Genant HK, Wu CY, van Kuijk C, Nevitt MC. Vertebral fracture assessment using a semiquantitative technique. *J Bone Miner Res* 1993; **8**: 1137–48.

2 Buckens CF, van der Graaf Y, Verkooijen HM, *et al.* Osteoporosis markers on low-dose lung cancer screening chest computed tomography scans predict all-cause mortality. *Eur Radiol* 2015; **25**: 132–9.

3 Burns JE, Yao J, Summers RM. Vertebral body compression fractures and bone density: Automated detection and classification on CT Images. *Radiology* 2017; **284**: 788–97.
